# The PRMT6/PARP1/CRL4B Complex Regulates the Circadian Clock and Promotes Breast Tumorigenesis

**DOI:** 10.1002/advs.202202737

**Published:** 2023-03-20

**Authors:** Tianshu Yang, Wei Huang, Tianyu Ma, Xin Yin, Jingyao Zhang, Miaomiao Huo, Ting Hu, Tianyang Gao, Wei Liu, Die Zhang, Hefen Yu, Xu Teng, Min Zhang, Hao Qin, Yunkai Yang, Baowen Yuan, Yan Wang

**Affiliations:** ^1^ Key Laboratory of Cancer and Microbiome State Key Laboratory of Molecular Oncology National Cancer Center/National Clinical Research Center for Cancer/Cancer Hospital Chinese Academy of Medical Sciences and Peking Union Medical College Beijing 100021 China; ^2^ Beijing Key Laboratory of Cancer Invasion and Metastasis Research Department of Biochemistry and Molecular Biology School of Basic Medical Sciences Capital Medical University Beijing 100069 China; ^3^ Key Laboratory of Immune Microenvironment and Disease (Ministry of Education) Department of Biochemistry and Molecular Biology School of Basic Medical Sciences Tianjin Medical University Tianjin 300070 China

**Keywords:** breast tumorigenesis, circadian rhythms, PARP1, PRMT6, the CRL4B complex

## Abstract

Circadian rhythms, as physiological systems with self‐regulatory functions in living organisms, are controlled by core clock genes and are involved in tumor development. The protein arginine methyltransferase 6 (PRMT6) serves as an oncogene in a myriad of solid tumors, including breast cancer. Hence, the primary aim of the current study is to investigate the molecular mechanisms by which the PRMT6 complex promotes breast cancer progression. The results show that PRMT6, poly(ADP‐ribose) polymerase 1 (PARP1), and the cullin 4 B (CUL4B)‐Ring E3 ligase (CRL4B) complex interact to form a transcription‐repressive complex that co‐occupies the core clock gene *PER3* promoter. Moreover, genome‐wide analysis of PRMT6/PARP1/CUL4B targets identifies a cohort of genes that is principally involved in circadian rhythms. This transcriptional‐repression complex promotes the proliferation and metastasis of breast cancer by interfering with circadian rhythm oscillation. Meanwhile, the PARP1 inhibitor Olaparib enhances clock gene expression, thus, reducing breast carcinogenesis, indicating that PARP1 inhibitors have potential antitumor effects in high‐PRMT6 expression breast cancer.

## Graphical abstract

Circadian rhythms are controlled by core clock genes and are involved in tumor development. It is demonstrated that protein arginine methyltransferase 6 (PRMT6), poly(ADP‐ribose) polymerase 1 (PARP1), and the cullin 4 B (CUL4B)‐Ring E3 ligase (CRL4B) complex interact to form a transcription‐repressive complex that co‐occupies the core clock gene PER3 promoter, promoting the proliferation and metastasis of breast cancer by interfering with circadian rhythm oscillation.

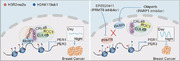

## 1 Introduction

Abnormal epigenetic modifications generally impact protein stability, enzymatic activity, or interactions, causing a multiplicity of systemic disorders, including cancer. Thus, targeting epigenetic regulation to impede cancer progression is the foundation for precision antitumor therapy. Indeed, abnormal histone methylation modification has been reported in many cancers, including breast cancers, liver cancers, and gastric cancers and is regulated by various proteins and transcription factors.

The protein arginine methyltransferase (PRMT) family, as writers of arginine methylation, catalyzes the addition of one or two methyl groups to the guanidine nitrogen atom of arginine to produce monomethyl arginine, asymmetric or symmetric dimethylarginine. In this way, PRMTs perturb gene transcription, DNA repair response, metabolic regulation, senescence, and chemotherapy resistance, thus inhibiting tumor progression.^[^ ^1^, ^2^, ^3^ ^]^ PRMT6 functions as a type I PRMT enzyme and is localized in the nucleus where it catalyzes the production of asymmetric dimethylarginine from histone substrates including H3R2, H4R3, H2AR3, and H2AR29,^[^ ^4^ ^]^ as well as nonhistone substrates including HIV Tat, RDA288, hnRNP D, CRAF, PTEN, and RCC1.^[^ ^5^, ^6^, ^7^ ^]^ Although PRMT6 is rarely mutated in tumors, it is highly expressed in various solid tumors, including breast tumors.^[^ ^8^ ^]^ In particular, an in‐depth exploration of the biological mechanisms underlying the functions of PRMT6 might serve to progress the development of cancer therapeutics.

In the present study, PRMT6 was found to interact directly with the nuclear localization signal (NLS) and BARD1 BRCA1 C‐terminal (BRCT) domains of the novel interacting protein poly(ADP‐ribose) polymerase 1 (PARP1). Meanwhile, PARP1 is the first PARP protein to be described as an ADP‐ribosyl transferase, whose main function is to participate in the DNA damage repair process through the homologous recombination (HR) and nonhomologous end joining (NHEJ) pathway. PARP1 alters other essential cellular events by modulating circadian rhythm, autophagy, fatty acid oxidation, inflammatory responses, as well as other pathways that affect disease progression.^[^ ^9^, ^10^ ^]^ Hence, understanding the non‐DNA repair‐related antitumor effects of PARP1 inhibitors may improve outcomes for refractory non‐BRAC mutated patients.

The circadian clock is an invisible “clock” that reflects intrinsic rhythmicity, and monitors various physiological functions. More specifically, at circadian time 0 (at dawn), clock circadian regulator (CLOCK) binds to aryl hydrocarbon receptor nuclear translocator like (*ARNTL*, i.e., *BMAL1*) to activate period circadian protein (PER) genes and cryptochrome (*CRY*) transcription. In contrast, at the end of the day (sunset), PER and CRY, in turn, repress *CLOCK* and *BMAL1* transcription. Degradation of PER and CRY via phosphorylation and ubiquitination relieves their transcriptional repression of *CLOCK* and *BMAL1*. This mechanism acts cooperatively with other auxiliary cycles to maintain core clock gene transcription levels throughout the 24 h oscillation period. In fact, approximately half of all mammalian genes exhibit rhythmic oscillatory expression. Although low expression of these core clock genes is common in malignancy, circadian rhythm abnormalities contribute to tumor proliferation and metastasis.^[^ ^11^, ^12^ ^]^ Indeed, circadian rhythm disorders have been implicated as potentially carcinogenic, presenting latent risk.^[^ ^13^ ^]^ Hence, examining circadian rhythm modulation and the synergistic relationships between clock and nonclock transcription factors may promote cancer therapy development.

The current study sought to investigate the molecular mechanisms by which the PRMT6 complex promotes breast cancer progression. We concluded that the PRMT6/PARP1/Cullin4B (CUL4B)‐Ring E3 ligase (CRL4B) complex transcriptionally represses *PER3* and promotes breast cancer proliferation, invasion, and metastasis. Furthermore, PARP1‐inhibition via Olaparib resulted in the dissolution of the PRMT6/PARP1/CRL4B complex, and strengthening the circadian rhythm amplitude and inhibiting breast cancer progression. Identification of novel regulators of circadian rhythms in cancer cells might provide insights into conceivable targets for cancer therapy.

## 2 Results

### 2.1 PRMT6 Acts as an Oncogene to Promote Breast Cancer Progression

**Figure 1:**
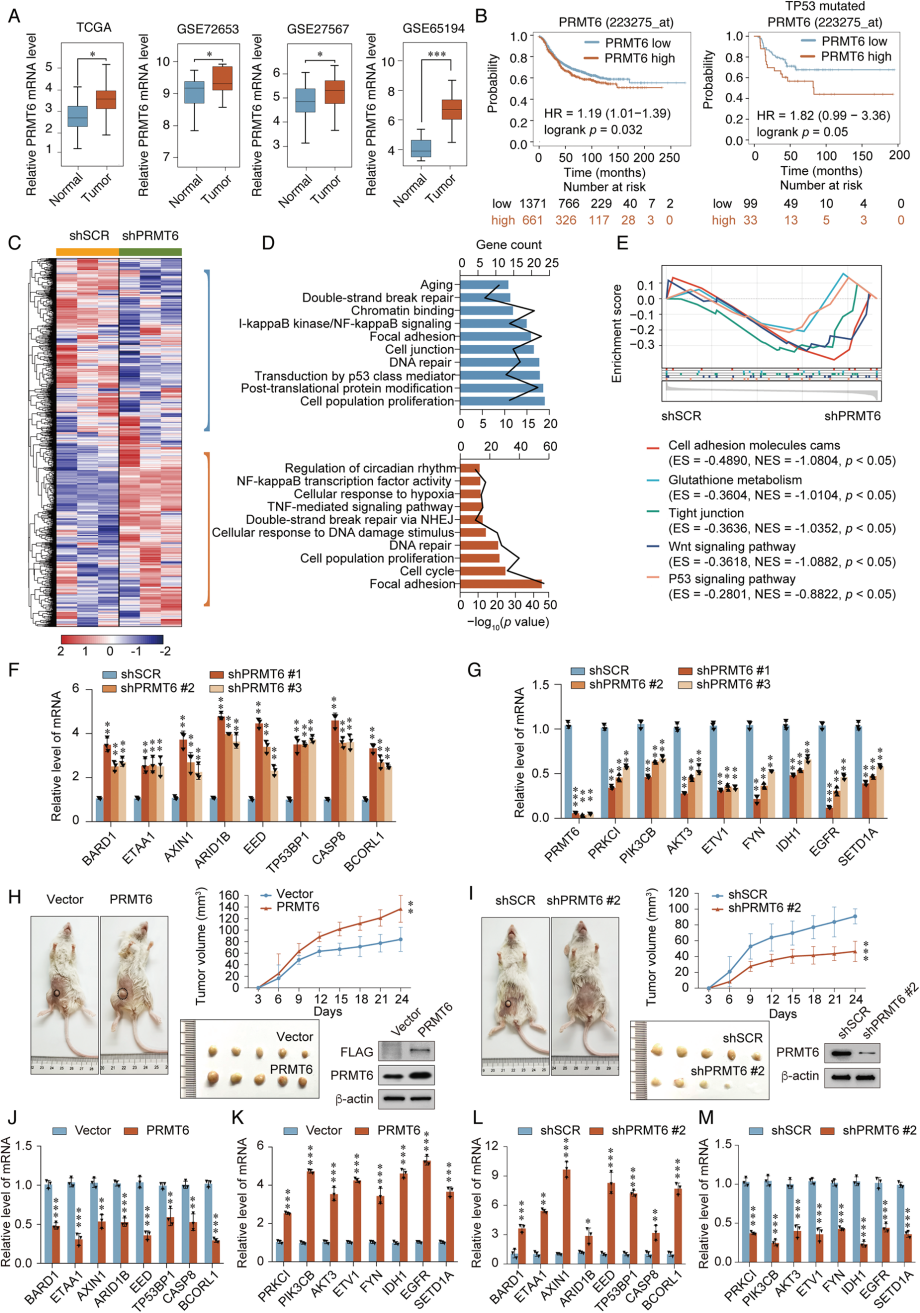
PRMT6 acts as an oncogene to promote breast cancer progression. A) Analysis of *PRMT6* mRNA expression in normal and breast cancer tissues within public datasets (TCGA BRAC, GSE72653, GSE27567, and GSE65194). Error bars represent the mean ± SD (*p <0.05, ** *p* < 0.01, *** *p* < 0.001). Two‐tailed unpaired *t*‐test. B) Kaplan–Meier survival analysis of PRMT6 expression in breast cancer or in *TP53*‐mutated breast cancer using the online tool Kaplan‐Meier plotter (http://kmplot.com/analysis/). “Number at risk” indicates the number of patients at risk at various times. C) Heat maps depicting the expression profile changes in control (shSCR) MDA‐MB‐231 cells and PRMT6‐knockdown (shPRMT6) MDA‐MB‐231 cells. D) GO enrichment analysis of differentially expressed genes in control cells and PRMT6‐ knockdown cells. Data analyzed using KOBAS (http://kobas.cbi.pku.edu.cn/). E) GSEA analysis of differentially expressed genes in control cells and PRMT6‐knockdown cells. GSEA analysis performed with Sangerbox tools. F,G) qRT‐PCR analysis of selected tumor suppressor genes and oncogenes in PRMT6‐knockdown (shPRMT6 #1, shPRMT 6 #2, and shPRMT6 #3) MDA‐MB‐231 cells. H,I) Tumor volumes in female NOD‐SCID mice (*n* = 5) following subcutaneous injection with MDA‐MB‐231 cells transfected with PRMT6 lentiviruses expression constructs or those carrying PRMT6‐specific shRNA. Western blot analysis of protein levels in separate tumors. J–M) qRT‐PCR analysis of selected tumor suppressor genes and oncogenes in *PRMT6*‐overexpressing and – knockdown tumors. F,G,J–M) Bars represent the mean ± SD of triplicate cell cultures (*p < 0.05, **p < 0.01, ***p < 0.001). Student's *t*‐test.

To explore the role of PRMT6 in the promotion of breast tumorigenesis, we evaluated *PRMT6* expression using RNA sequencing (RNA‐seq) datasets from The Cancer Genome Atlas (TCGA) and Gene Expression Omnibus (GEO). Results indicate that *PRMT6* is upregulated in breast cancer, compared with normal tissues; higher levels of *PRMT6* were correlated with poor overall survival in patients with breast cancer (**Figure** **1**A,B), confirming that PRMT6 expression is associated with breast cancer development, as previously reported.^[^ ^14^ ^]^

PRMT6‐knockdown MDA‐MB‐231 cells were established using lentivirus‐delivered short hairpin RNA (shRNA) for RNA‐seq analysis to examine how PRMT6 promotes breast cancer growth. In total, 1551 genes were upregulated, while 771 were downregulated relative to the control (Figure 1C). Gene Ontology (GO) analysis further revealed that the downregulated genes were enriched in pathways associated with aging, cell junction, DNA repair, and the p53 mediator, whereas the upregulated genes were associated with circadian rhythm, hypoxia, DNA repair, and cell cycle (Figure 1D). Additionally, Gene Set Enrichment Analysis (GSEA) of differentially expressed PRMT6 target genes revealed enrichment in cell adhesion, glutathione metabolism, WNT signaling, and the p53 signaling pathway (Figure 1E). These results imply that PRMT6 participates in breast cancer progression via essential cellular processes.

To elucidate the role of PRMT6 in breast cancer, we verified the mRNA expression levels of potential tumor suppressor genes (TSGs) and oncogenes. Consistent with the RNA‐seq results, the expression of potential TSGs, namely, *BARD1*, *ETAA1*, *AXIN1*, *ARID1B*, *EED*, *TP53BP1*, *CASP8*, and *BCORL1* were augmented, whereas that of potential oncogenes, including *PRKCI*, *PIK3CB*, *AKT3*, *ETV1*, *FYN*, *IDH1*, *EGFR*, and *SETD1A* were diminished in PRMT6‐depleted cells (Figure 1F,G). To further confirm our results in vivo, PRMT6‐knockdown or ‐overexpressing MDA‐MB‐231 cells were injected subcutaneously into female NOD‐SCID mice (*n* = 5). Overexpression of PRMT6 caused significant augmentation of subcutaneous tumor growth (Figure 1H and Figure S1A, Supporting Information), whereas PRMT6 knockdown caused the opposite result, relative to the control (Figure 1I and Figure S1B, Supporting Information). Additionally, to validate the RNA‐seq results in vivo, the expression of the potential TSGs and oncogenes were assessed in breast tumor tissues. Consistent with the in vitro results, the expression of TSGs was reduced in PRMT6‐overexpressing tumors and enhanced in PRMT6‐knockdown tumors (Figure 1J,L), the mRNA levels of oncogenes exhibited the opposite effect (Figure 1K,M). These in vitro and in vivo results suggest that PRMT6 contributes to breast tumor growth by coordinating with key tumor regulatory genes in vitro and in vivo.

### 2.2 PRMT6 Modulates DNA Repair to Improve Breast Cancer Cell Proliferation and Invasion

**Figure 2:**
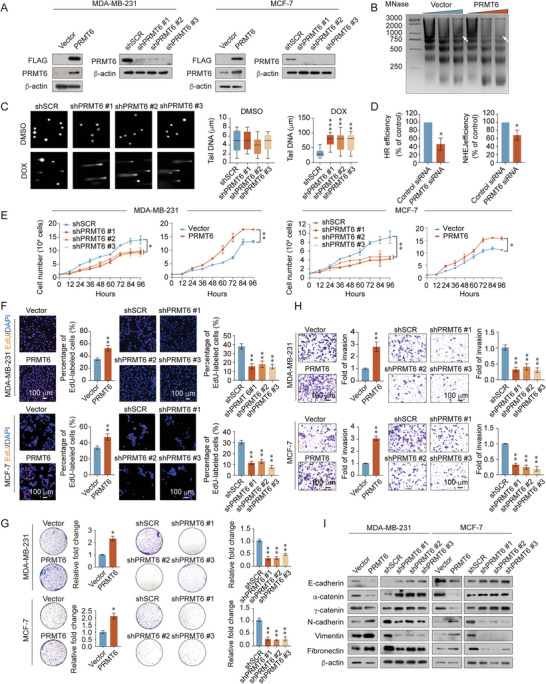
PRMT6 modulates DNA repair to improve breast cancer cell proliferation and invasion. A) Establishment of the stably overexpressing PRMT6, knockdown (shPRMT6 #1, shPRMT6 #2, and shPRMT6 #3), and control, MDA‐MB‐231 and MCF‐7 cell lines. B) MNase digestion assay in PRMT6‐overexpressing MDA‐MB‐231 cells. MNase concentration: 1, 5, and 10 units. C) PRMT6 knockdown (shPRMT6 #1, shPRMT6 #2, and shPRMT6 #3) MDA‐MB‐231 cells treated with DOX or dimethyl sulfoxide (DMSO) for 2 h. DNA damage in these cells was measured using an alkaline comet assay (25 cells), and tail DNA is shown. D) Homologous repair (HR) efficiency determined via FACS, for PRMT6‐knockdown DR‐GFP U2OS cells (left panel). Nonhomologous end joining (NHEJ) efficiency, determined via FACS, for PRMT6‐knockdown EJ5‐U2OS cells (right panel). E) Cell proliferation curves, F) EdU assay, G) colony formation assay, H) transwell invasion assay, and I) western blot analysis of EMT markers expression, in stably overexpressing‐PRMT6 and PRMT6‐knockdown (shPRMT6 #1, shPRMT6 #2, and shPRMT6 #3) MDA‐MB‐231 and MCF‐7 cells. C–H) Bars represent the mean ± SD of three independent experiments (*p < 0.05, **p < 0.01, ***p < 0.001). Student's *t*‐test. shSCR, control scrambled shRNA.

Although PRMT6 has been shown to participate in the DNA repair process, the underlying response mechanism remains unclear. MNase digestion assays were conducted to investigate whether PRMT6 affects chromatin condensation, and to clarify the involvement of PRMT6 in DNA repair. Prior western blotting of shRNA and plasmid verified their respective knockdown or overexpression efficacy in MDA‐MB‐231 and MCF‐7 cells (**Figure** **2**A and Figure S1C, Supporting Information). The MNase assay results further revealed that PRMT6 gain of function led to augmentation of chromatin accessibility (Figure 2B and Figure S1D, Supporting Information).

Next, alkaline comet assays were performed to estimate overall DNA damage in *PRMT6*‐knockdown or ‐overexpressing MDA‐MB‐231 cells treated with the DNA‐damaging reagent doxorubicin (DOX). PRMT6 deletion caused massive DNA damage, with increased tail moment lengths, whereas PRMT6 gain‐of‐function cells exhibited minimal DNA damage compared with that of the control (Figure 2C and Figure S1E, Supporting Information). HR and NHEJ reporter assays were performed to elucidate the role of PRMT6 in DNA repair. Results revealed that PRMT6 overexpression significantly increased HR and NHEJ efficiency, while PRMT6 deletion induced the reverse effect, indicating that PRMT6 might participate in DNA repair by inducing HR and NHEJ responses (Figure 2D and Figure S1F, Supporting Information).

Additionally, growth curve assays, 5‐ethynyl‐2'deoxyuridine (EdU) incorporation assays, colony formation assays, and Transwell assays were conducted to evaluate the cellular proliferation, migration, and invasive capacity of breast cancer cells and to further verify the tumorigenic function of PRMT6 in vitro. PRMT6 gain‐of‐function cells exhibited augmented proliferation and colony numbers, while PRMT6 depletion decreased these effects in the triple‐negative cell line MDA‐MB‐231 and luminal breast cancer cell line MCF‐7 cells (Figure 2E–G). Moreover, PRMT6 overexpression led to a significant increase in the migration and invasive potential of breast cancer cells, while PRMT6 deletion eliminated metastatic capacity (Figure 2H and Figure S1G, Supporting Information). Considering that epithelial‐mesenchymal transition (EMT) is the main pathway involved in invasion and metastasis leading to tumor progression,^[^ ^15^ ^]^ the levels of EMT markers were assessed to clarify the molecular role of PRMT6 in breast cancer metastasis. Western blot results revealed that PRMT6 significantly decreased the expression of epithelial cell markers, including E‐cadherin, *α*‐catenin, and *γ*‐catenin, while increasing that of mesenchymal cell markers, namely, N‐cadherin, vimentin, and fibronectin. In contrast, PRMT6 depletion had the opposite effects on EMT marker expression (Figure 2I and Figure S1H, Supporting Information). Meanwhile, ER status did not affect the role of PRMT6 in breast cancer. Collectively, these phenotypic results suggest that PRMT6 represents a novel DNA repair factor required to sustain breast cancer progression.

### 2.3 PRMT6 Physically Interacts with PARP1 and the CRL4B Complex

**Figure 3:**
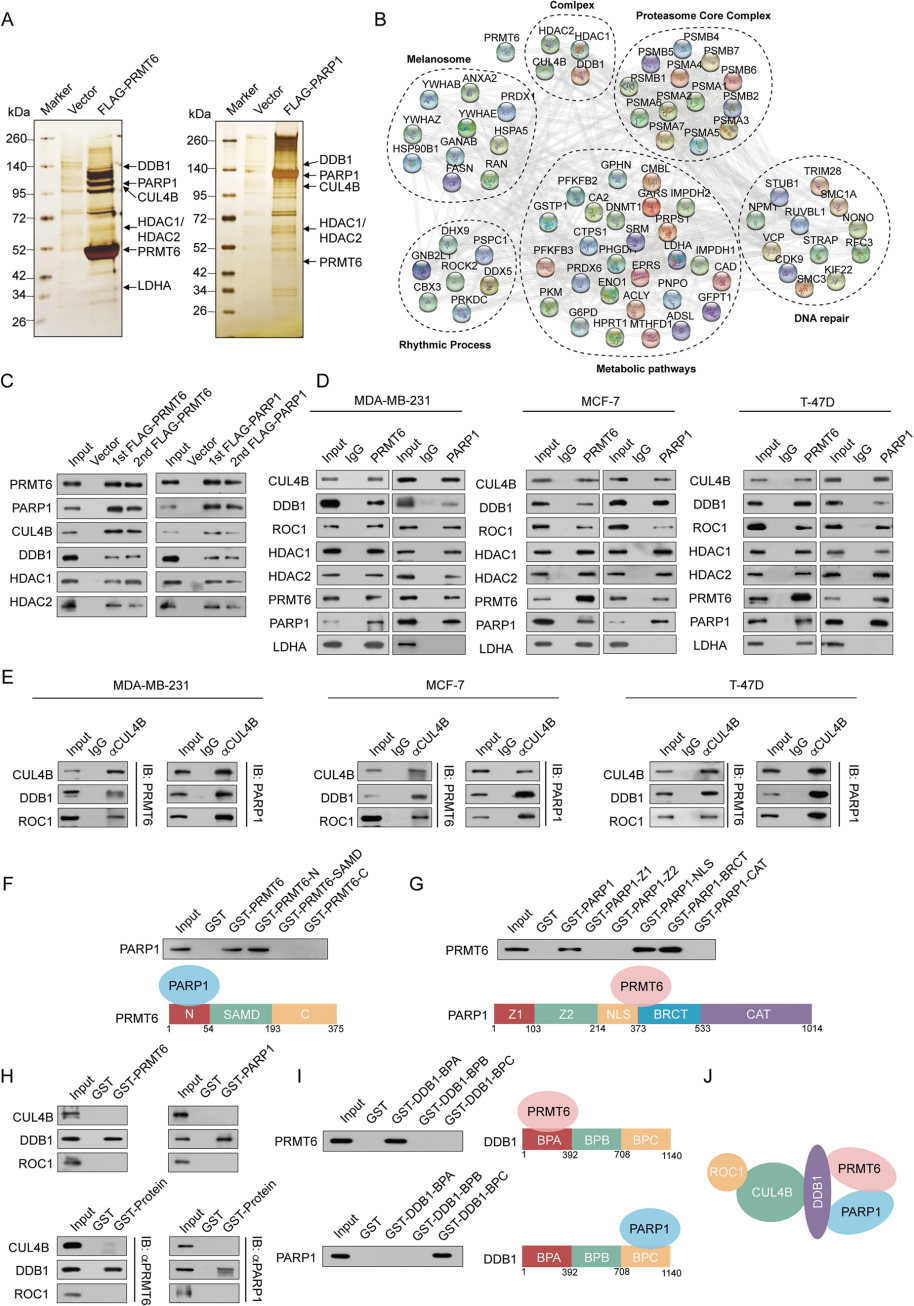
PRMT6 physically interacts with PARP1 and the CRL4B complex. A) Immunoaffinity purification and mass spectrometry analysis of PRMT6 and PARP1 containing protein complexes. PRMT6 and PARP1 protein bands were retrieved and analyzed using mass spectrometry. B) STRING network plot depicting relationships among overlapping interacting proteins between PRMT6 and PARP1 containing protein complexes. C) Western blot analysis of the purified fractions from HEK‐293T cells. D,E) Association of PRMT6 with PARP1 and CRL4B complex in MDA‐MB‐231, MCF‐7, and T‐47D cells. Whole‐cell lysates were prepared, and coimmunoprecipitation (Co‐IP) was performed. F) GST pull‐down assays with GST‐fused PRMT6 N‐terminal domain (N), SAMD, or C‐terminal domain (C) and in vitro transcribed/translated PARP1. G) GST pull‐down assays with GST‐fused PARP1 zinc finger 1 (Z1), zinc finger 2 (Z2), nuclear localization signal (NLS), BRCA1 C terminus (BRCT), or CAT and in vitro transcribed/translated PRMT6. H) GST pull‐down assays with bacterially expressed GST‐fused proteins and in vitro transcribed/translated proteins. I) GST pull‐down assays with three GST‐fused DDB1 propeller domains (BPA, BPB, or BPC) and in vitro transcribed/translated PRMT6 or PARP1. J) Schematic diagram depicting molecular interactions between PRMT6, PARP1, and the CRL4B complex.

Protein mass spectrometry was employed to screen interacting proteins in PRMT6‐overexpressing HEK‐293T cells, to determine whether PRMT6 contributes to carcinogenesis by forming complexes with chromatin modifiers. The detailed results are shown in Tables S1 and S2 in the Supporting Information. PRMT6 was copurified with PARP1, CUL4B, DDB1, HDAC1, HDAC2, and LDHA (**Figure** **3**A). Moreover, given that PARP1 is the key ADP‐ribosyl transferase in the DNA repair process, and repairs DNA damage by inducing the HR and NHEJ pathways, with a function that is similar to that of PRMT6, proteins that interact with PARP1 were also screened. Analysis revealed that the purified proteins that interact with PARP1 (CUL4B, DDB1, HDAC1, HDAC2, and PRMT) overlapped substantially with PRMT6 (Figure 3A). The overlapping interacting proteins were then analyzed by STRING network, to discover the latent functional relationship between PARP1 and PRMT6. The latent PRMT6 and PARP1 complex was found to potentially participate in proteasome core complex formation, melanosome synthesis, rhythmic processes, metabolic pathways, and DNA repair (Figure 3B). PRMT6, PARP1, CUL4B, DDB1, HDAC1, and HDAC2 were verified using the column eluates from the PRMT6 or PARP1 mass spectrometry assays (Figure 3C). The co‐immunoprecipitation (Co‐IP) assay results confirmed the combination of PRMT6 and PARP1, further demonstrating that PRMT6 and PARP1 interact with the CRL4B complex including CUL4B, DDB1, and ROC1 in MDA‐MB‐231, MCF‐7, and T‐47D cells (Figure 3D,E and Figure S2A, Supporting Information). Further elucidation of the PRMT6/PARP1 interaction domains via glutathione S‐transferase (GST) pull‐down assays, revealed that PRMT6 N‐terminal sequences, and the PARP1 NLS motif and BRCT domain, are required for the direct interaction of these proteins (Figure 3F,G and Figure S2B,C, Supporting Information). Moreover, in vitro GST pull‐down assays revealed direct binding between the DDB1 BPA domain and PRMT6, as well as between the DDB1 BPC domain and PARP1 (Figure 3H,I and Figure S2D, Supporting Information). In contrast, CUL4B and ROC1 did not interact directly with PRMT6 or PARP1. Hence, DDB1 might capture PRMT6 and PARP1 by combining them with the PRMT6/PARP1/CRL4B complex (Figure 3H,J).

The arginine of the target protein RCC1 can be methylated via interacting with the PRMT6 N‐terminal.^[^ ^7^ ^]^ Thus, Co‐IP assays were performed, with antibodies against the pan‐monomethylation, pan‐symmetry dimethylation, and pan‐asymmetry dimethylation of arginine, to confirm that PARP1 arginine becomes methylated in vivo. Results revealed that PARP1 arginine was monomethylated, pan‐symmetry demethylated, and pan‐asymmetry dimethylated (Figure S2E, Supporting Information). To confirm the hypothesis that PRMT6 methylates PARP1, we performed in vitro methylation assays; however, PRMT6 was unable to asymmetrically dimethylate PARP1 arginine (Figure S2F, Supporting Information). Considering that the PARP1 BRCT domain mediates protein interactions via post‐translational modification poly(ADP‐ribosyl)ation (PARylation),^[^ ^16^ ^]^ do determine whether PARP1 serves as the recognition site of PRMT6‐generated histone arginine methylation, we performed GST pull‐down assays using GST‐PARP1, GST‐PRMT6, and purified histone octamers. PRMT6‐generated H3R2me2a was not recognized by PARP1 (Figure S2G, Supporting Information). We then characterized whether PRMT6 or DDB1 can be PARylated by PARP1 using Co‐IP assay with an anti‐PAR antibody. However, results showed that PRMT6 and DDB1 were not among the PARylated proteins (Figure S2H, Supporting Information). Hence, PRMT6 and PARP1 did not post‐translationally modify each other.

**Figure 4:**
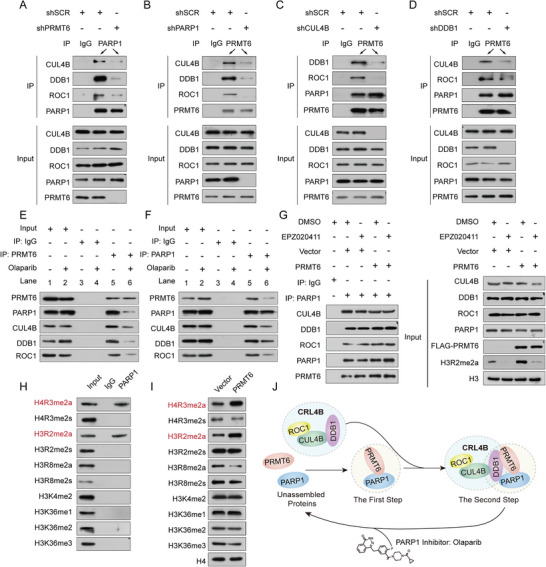
Assembly sequence of the PRMT6/PARP1/CRL4B complex. A) Lysates from PRMT6‐knockdown MDA‐MB‐231 cells immunoprecipitated with control IgG or an anti‐PARP1 antibody, followed by immunoblotting with indicated antibodies. B) Lysates from PARP1‐knockdown MDA‐MB‐231 cells immunoprecipitated with control IgG or anti‐PRMT6 antibody, followed by immunoblotting with indicated antibodies. C) Lysates from CUL4B‐knockdown MDA‐MB‐231 cells immunoprecipitated with control IgG or an anti‐PRMT6 antibody, followed by immunoblotting with indicated antibodies. D) Lysates from DDB1‐knockdown MDA‐MB‐231 cells immunoprecipitated with control IgG or an anti‐PRMT6 antibody, followed by immunoblotting with indicated antibodies. E) Lysates from MDA‐MB‐231 cells with Olaparib treatment immunoprecipitated with control IgG or an anti‐PRMT6 antibody, followed by immunoblotting with indicated antibodies. F) Lysates from MDA‐MB‐231 cells with Olaparib treatment immunoprecipitated with control IgG or an anti‐PARP1 antibody, followed by immunoblotting with indicated antibodies. G) Lysates from PRMT6‐overexpressing PRMT6 MDA‐MB‐231 cells treated with EPZ020411 immunoprecipitated with control IgG or an anti‐PARP1 antibody, followed by immunoblotting with indicated antibodies. H) Lysates from MDA‐MB‐231 cells immunoprecipitated with control IgG or an anti‐PARP1 antibody, followed by immunoblotting with indicated antibodies. I) Western blot analysis of the indicated chromatin modifications in stably overexpressing PRMT6 MDA‐MB‐231 cells. J) Schematic diagram depicting the assembled sequence of the PRMT6/PARP1/CRL4B complex.

Next, we depicted the assembly of PRMT6, PARP1, and the CRL4B complex using Co‐IP assays. The interactions between PARP1 and CRL4B components were found to be PRMT6‐dependent; likewise, the interaction between PRMT6 and CRL4B components was PARP1‐dependent (**Figure** **4**A,B). In contrast, CUL4B and DDB1 did not affect the interaction between PRMT6 and PARP1 (Figure 4C,D). Co‐IP assays with the PARP1 inhibitor Olaparib were then conducted to determine whether PRMT6/PARP1/CRL4B complex formation requires PARP1 enzymatic activity. Olaparib treatment did not impact the expression of PRMT6, PARP1, or the CRL4B complex (Figure S2I, Supporting Information). Moreover, inhibition of PARP1 activity by Olaparib also impeded cooperation between PARP1/PRMT6 and the CRL4B complex (Figure 4E,F).

Then, we sought to determine whether PRMT6/PARP1/CRL4B complex formation requires PRMT6 enzymatic activity by performing Co‐IP assays with the PRMT6 inhibitor EPZ020411. Unlike the PARP1 inhibitor, the PRMT6 inhibitor EPZ020411 did not affect the assembly of PRMT6, PARP1, and the CRL4B complex (Figure 4G and Figure S2K, Supporting Information). These results support the hypothesis that the PRMT6/PARP1/CRL4B complex may initially require PRMT6 and PARP1 to assemble before the complex is recognized by DDB1 to form a new complex with the CRL4B complex (Figure 4J). Considering how the PRMT6/PARP1/CRL4B complex is assembled, we assessed whether PARP1 is associated with recognition of PRMT6 histone arginine methylation. Based on Co‐IP assay results, PARP1 co‐immunoprecipitated with H4R3me2a and H3R2me2a, which are mediated by PRMT6 in HEK‐293T and MDA‐MB‐231 cells (Figure 4H,I and Figure S2J,L, Supporting Information). Taken together, these findings describe a role for PARP1 in recognizing PRMT6‐mediated histone modifications by binding to PRMT6.

### 2.4 The PRMT6/PARP1/CRL4B Complex Exerts Transcriptional Activity

**Figure 5:**
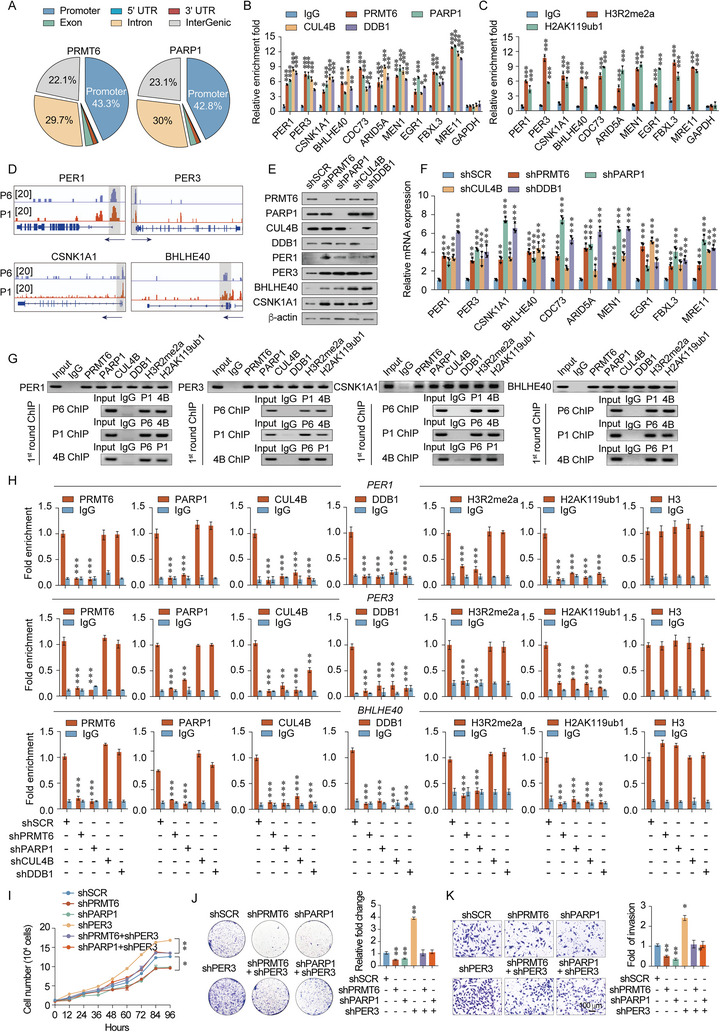
The PRMT6/PARP1/CRL4B complex exerts transcriptional activity. A) ChIP‐seq analysis of PRMT6 and PARP1 chromosome features. B,C) Verification of ChIP‐seq results using qChIP analysis of genes identified in MDA‐MB‐231 cells. Results are represented as fold change over control; *GAPDH* served as the internal control. D) Binding profiles of PRMT6 and PARP1 on the representative target genes *PER1, PER3, CSNK1A1*, and *BHLHE40*. P1, PARP1; P6, PRMT6. E) Western blot analysis of indicated proteins in the stable PRMT6‐knockdown, PARP1‐knockdown, CUL4B‐knockdown, and DDB1‐knockdown MDA‐MB‐231 cells. F) mRNA levels of indicated genes in the PRMT6‐knockdown, PARP1‐knockdown, CUL4B‐knockdown, and DDB1‐knockdown MDA‐MB‐231 cells. G) ChIP and Re‐ChIP analysis in MDA‐MB‐231 cells with indicated antibodies. P1, PARP1; P6, PRMT6; 4B, CUL4B. H) Recruitment of indicated proteins to *PER1*, *PER3*, *BHLHE40*, and *CSNK1A1* promoters in MDA‐MB‐231 cells after transfection with control shRNA (shSCR) or shRNAs targeting PRMT6, PARP1, CUL4B, or DDB1. Purified rabbit IgG was used as a negative control. I) Cell proliferation curves for control, PRMT6‐knockdown, PARP1‐knockdown, and PER3‐knockdown MDA‐MB‐231 cells. J) Colony formation assays for control, PRMT6‐knockdown, PARP1‐knockdown, and PER3‐knockdown MDA‐MB‐231 cells. K) Transwell invasion assays for control, PRMT6‐knockdown, PARP1‐knockdown, and PER3‐knockdown MDA‐MB‐231 cells. B,C,F,H,J,K) Bars represent the mean ± SD of three independent experiments (*p < 0.05, **p < 0.01, ***p < 0.001). Student's *t*‐test.

Given that our findings exclude post‐translational modification between PRMT6 and PARP1, the PRMT6/PARP1/CRL4B complex might exert biological functions by transcriptionally regulating target genes. To identify the genome‐wide transcriptional target genes of the PRMT6/PARP1/CRL4B complex, we performed chromatin immunoprecipitation‐based deep sequencing (ChIP‐seq) with anti‐PRMT6 or anti‐PARP1 antibodies in MDA‐MB‐231 cells. Results revealed 3763 PRMT6‐specific and 4672 PARP1‐specific binding promoters (**Figure** **5**A and Figure S3A, Supporting Information). Notably, PRMT6 and PARP1 had similar binding motifs, providing further evidence of the physical binding between these two proteins (Figure S3B, Supporting Information). We then assessed the 2522 overlapping gene promoters targeted by the PRMT6/PARP1 complex and 1833 overlapping gene promoters targeted by the PRMT6/PARP1/CRL4B complex. The 1833 promoters were systematized by the gene ontology pathway using KOBAS (Figure S3C, Supporting Information). These overlapping promoters were classified into diverse GO pathways, the top 10 of which included pathways critical to tumor progression, namely, the Wnt signaling pathway, DNA repair, circadian regulation, and cell cycling (Figure S3C,D, Supporting Information). Additionally, the overlapping genes between the RNA‐seq and ChIP‐seq were analyzed to mine deeper data. Results indicated that the GO pathway‐enriched classifications included metabolic pathways, cell cycle, the p53 pathway, cellular senescence, circadian regulation, and the DNA repair pathway (Figure S3E, Supporting Information). Quantitative ChIP (qChIP) analysis further confirmed that the promoters of the target genes associated with four GO pathways (Wnt signaling, DNA repair, circadian regulation, and cell cycle) were strongly enriched. These genes were *PER1*, *PER3*, *CSNK1A1*, *BHLHE40*, *CDC73*, *ARID5A*, *MEN1*, *EGR1*, *FBXL3*, and *MRE11* (Figure 5B,C). Moreover, the significant enrichment of PRMT6 and PARP1 was mapped to the *PER1*, *PER3*, *BHLHE40*, and *CSNK1A1* promoters (Figure 5D).

Given that PRMT6, PARP1, and the CRL4B complex target these promoters, the mRNA and protein expression levels for each target gene were assessed following shRNA‐mediated deletion of PRMT6, PARP1, CUL4B, and DDB1 in MDA‐MB‐231 cells. Knockdown of PRMT6, PARP1, CUL4B, or DDB1 increased the expression of *PER1*, *PER3*, *CSNK1A1*, and *BHLHE40* at the mRNA and protein levels, while increasing the mRNA levels of *CDC73*, *ARID5A*, *MEN1*, *EGR1*, *FBXL3*, and *MRE11* (Figure 5E,F and Figure S3F,G, Supporting Information). Subsequent ChIP/Re‐ChIP assays revealed that PRMT6, PARP1, and CUL4B occupied the promoters of *PER1*, *PER3*, *CSNK1A1*, and *BHLHE40*, as a single protein complex (Figure 5G). However, qChIP assay findings indicated that CUL4B or DDB1 loss‐of‐function did not affect the occupation of *PER1*, *PER3*, *CSNK1A1*, and *BHLHE40* promoters by PRMT6 and PARP1, while PRMT6 or PARP1 loss‐of‐function scarcely diminished the occupation of these target promoters by CUL4B and DDB1. Meanwhile, PRMT6, PARP1, CUL4B, or DDB1 deletion homogenously reduced the association between H2AK119ub1 and *PER1*, *PER3*, *CSNK1A1*, and *BHLHE40* promoters. Conversely, CUL4B or DDB1 deletion did not interfere with the association between H3R2me2a and any of the target promoters (Figure 5H and Figure S3J, Supporting Information). Hence, PRMT6 and PARP1 likely act as the principal components of the novel complex with the CRL4B complex that functionally exercises transcriptional repression of these target genes.

We then sought to further determine whether the PRMT6/PARP1/CRL4B complex exerts an essential role in cell proliferation and invasion via transcriptionally repressing target genes. To this end, the expression of PRMT6, PARP1, and PER3 in the constructed cell models was validated using western blotting (Figure S3H,I, Supporting Information). The growth curve and colony formation assay results revealed that inhibition of PER3 nearly rescued cellular proliferation repressed by the deletion of PRMT6 and PARP1 (Figure 5I,J). In addition, the Transwell assay results revealed similar salvage effects in terms of invasive ability following PER3 knockdown. Moreover, knockdown of PRMT6 and PARP1 significantly impeded breast cancer cell invasiveness, whereas constraining PER3 expression restored it to normal levels (Figure 5K). Thus, formation of the PRMT6/PARP1/CRL4B complex is vital in promoting proliferative and tumorigenic effects.

### 2.5 PRMT6/PARP1/CRL4B Complex Formation Functionally Interrupts the Tumor‐Autonomous Circadian Clock

**Figure 6:**
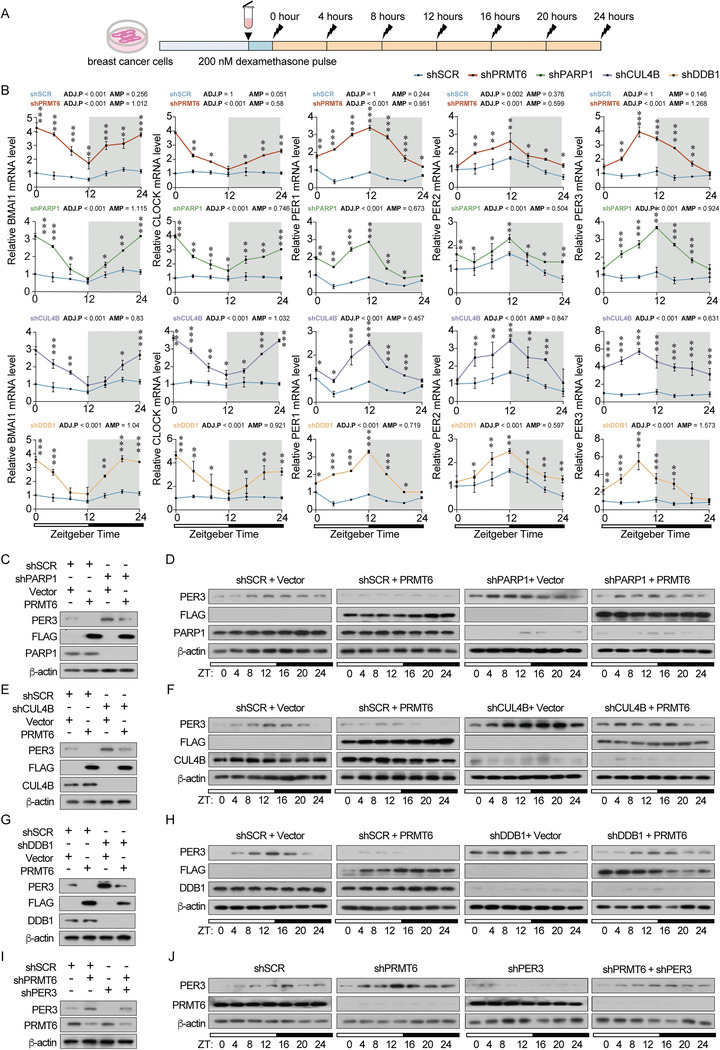
PRMT6/PARP1/CRL4B complex formation functionally interrupts the tumor‐autonomous circadian clock. A) Schematic of the experimental schedule to perform time‐series protein assays in breast cancer cells. Collection of cells at 4 h interval from 0 to 24 h after 24 h of dexamethasone synchronization. B) qRT‐PCR analysis of *BMAI1*, *CLOCK*, *PER1*, *PER2*, and *PER3* in control, PRMT6‐knockdown, PARP1‐knockdown, CUL4B‐knockdown, and DDB1‐knockdown MDA‐MB‐231 cells. The mRNA expression levels were analyzed by the JTK‐CYCLE method. ADJ.P, adjusted minimal *p*‐values; AMP, amplitude. Bars represent the mean ± SD of three independent experiments (*p < 0.05, **p < 0.01, ***p < 0.001) compared with shSCR cells 0 h. Two‐way ANOVA. C) PER3, PARP1, and FLAG protein abundance in MDA‐MB‐231 cells transfected with control shRNA (shSCR) or shRNAs targeting PARP1 with or without stable overexpression of PRMT6. D) Western blot analysis of the indicated proteins in PARP1‐knockdown MDA‐MB‐231 cells with or without stable overexpression of PRMT6. E) PER3, CUL4B, and FLAG protein abundance in MDA‐MB‐231 cells transfected with control shRNA (shSCR) or shRNAs targeting CUL4B with or without stable overexpression of PRMT6. F) Western blot analysis of indicated proteins in CUL4B‐knockdown MDA‐MB‐231 cells with or without stable overexpression of PRMT6. G) PER3, DDB1, and FLAG protein abundance in MDA‐MB‐231 cells transfected with control shRNA (shSCR) or shRNAs targeting DDB1 with or without stable overexpression of PRMT6. H) Western blot analysis of indicated proteins in DDB1‐knockdown MDA‐MB‐231 cells with or without stable overexpression of PRMT6. I) PER3 and PRMT6 protein abundance in MDA‐MB‐231 cells transfected with control shRNA (shSCR) or shRNAs targeting PER3 and/or PRMT6. J) Western blot analysis of indicated proteins in PER3‐knockdown MDA‐MB‐231 cells with or without PRMT6 knockdown. D,F,H,J) ZT, Zeitgeber Time.

ChIP‐seq analysis revealed that the PRMT6/PARP1/CRL4B complex transcriptionally repressed the expression of core clock genes expression. Thus, we synchronized the cells via serum starvation for 24 h, followed by treatment with dexamethasone for 2 h to monitor clock gene expression oscillation (**Figure** **6**A). PRMT6, PARP1, CUL4B, or DDB1 loss‐of‐function resulted in robust oscillation of *BMAI1*, *CLOCK*, *PER1*, *PER2*, and *PER3*, however, that of *CRY1* and *CRY2* was similar to the control cells (Figure 6B and Figure S4A, Supporting Information). Thus, the absence of the PRMT6/PARP1/CRL4B complex may modulate circadian rhythm oscillation. To strengthen this conjecture, we used time‐series protein assays to investigate the effect of PRMT6/PARP1/CRL4B complex assembly on circadian rhythms. PRMT6 gain‐of‐function cells exhibited a decrease in the oscillation of PER3 expression, primarily due to complex formation with PARP1 and the CRL4B complex. Moreover, interrupting complex assembly by deleting PARP1, CUL4B, or DDB1 caused PER3 oscillation in PRMT6‐overexpressing cells to the baseline level of control cells (Figure 6C–H and Figure S4B, Table S3, Supporting Information). Moreover, PRMT6 altered the amplitude of PER3 oscillations by transcriptionally repressing *PER3*; subsequent simultaneous withdrawal of PER3 in PRMT6 loss of function cells counteracted this transcriptional repression (Figure 6I,J and Figure S4B, Table S3, Supporting Information). These results emphasize that the PRMT6/PARP1/CRL4B complex interferes with circadian rhythms via transcriptional repression of PER3 oscillation.

### 2.6 PARP1 Inhibitor Olaparib Affects the Circadian Rhythm

**Figure 7:**
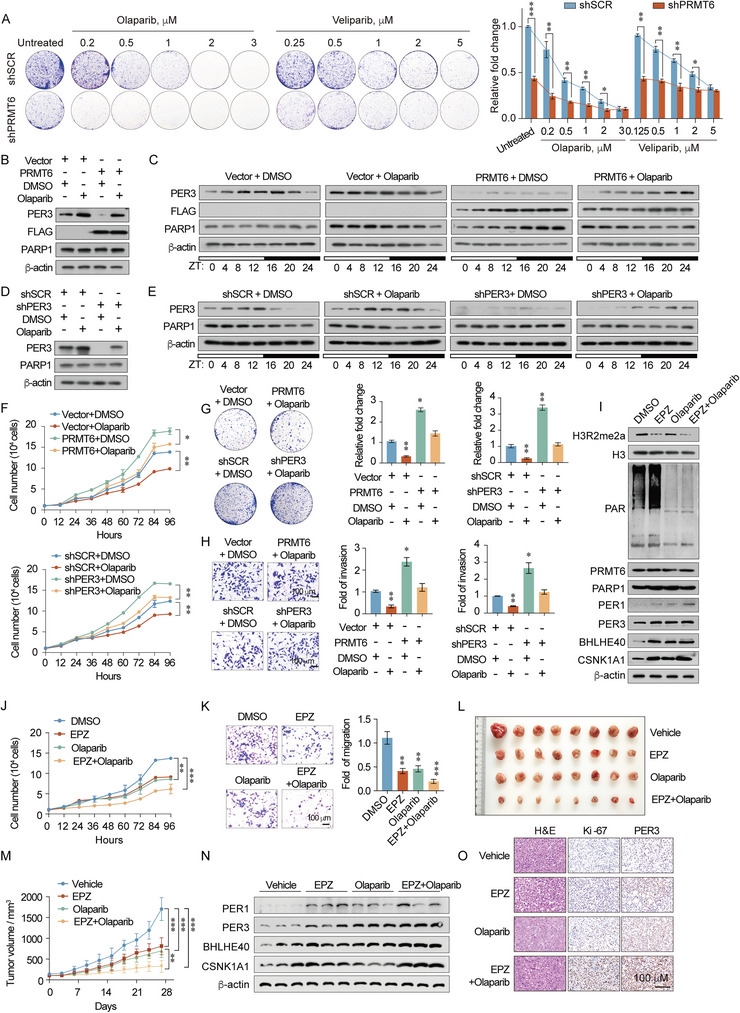
PARP1 inhibitor Olaparib affects the circadian rhythm. A) MDA‐MB‐231 cells transfected with control shRNA (shSCR) or shRNAs targeting PRMT6 and treated with different doses of Olaparib or Veliparib. Representative images (left), and quantification of colony formation (right), from biological triplicate experiments are shown. B) PER3, FLAG, and PARP1 abundance in MDA‐MB‐231 cells overexpressing PRMT6 treated with Olaparib or DMSO. C) Western blot analysis of indicated proteins in MDA‐MB‐231 cells overexpressing PRMT6 following treatment with Olaparib or DMSO. ZT, Zeitgeber Time. D) PER3 and PARP1 abundance in MDA‐MB‐231 cells transfected with control shRNA (shSCR) or shRNAs targeting PER3 following treatment with Olaparib or DMSO. E) Western blot analysis of indicated proteins in PER3‐knockdown MDA‐MB‐231 cells treated with Olaparib or DMSO. ZT, Zeitgeber Time. F) Cell proliferation curves for control and PRMT6‐overexpressing MDA‐MB‐231 cells treated with Olaparib or DMSO (upper panel). Cell proliferation curves for control and PER3‐knockdown MDA‐MB‐231 cells treated with Olaparib or DMSO (lower panel). G) Colony formation assays for control and PRMT6‐overexpressing or ‐knockdown MDA‐MB‐231 cells treated with Olaparib or DMSO. H) Transwell invasion assays for control, and PRMT6‐overexpressing or ‐knockdown MDA‐MB‐231 cells treated with Olaparib or DMSO. I) Western blotting analysis of MDA‐MB‐231 cells treated with Olaparib and/or EPZ020411 with antibodies against indicated proteins. EPZ, EPZ020411. J) Cell proliferation curves of MDA‐MB‐231 cells treated with Olaparib and/or EPZ020411. K) Transwell invasion assays of MDA‐MB‐231 cells treated with Olaparib and/or EPZ020411. L,M) MDA‐MB‐231 cells were subcutaneously inoculated into the abdominal mammary fat pads of 6 weeks old female NOD‐SCID mice (*n* = 8), and tumor volumes were measured weekly. When the tumor size was ≈100 mm^3^, treatment with Olaparib or/and EPZ020411 was initiated. N) Western blotting analysis of 12 independent tumor tissues harvested from animals and treated with antibodies against indicated proteins. EPZ, EPZ020411. O) H&E staining of tumor tissues. IHC staining of Ki‐67 and PER3 in tumor tissues. Representative photos of three specimens are shown. Scale bars, 100 µm. A,F,G,H,J,K) Bars represent the mean ± SD of three independent experiments (*p < 0.05, **p < 0.01, ***p < 0.001). Student's *t*‐test. M) Data are presented as mean ± SEM. (**p < 0.01, ***p < 0.001). Student's *t*‐test.

To this point, we have demonstrated that PARP1 enzymatic activity is necessary for assembly of the PRMT6/PARP1/CRL4B complex. Further long‐term colony formation analysis revealed that the PARP1 inhibitor, Olaparib, was more efficient than Veliparib at restricting cellular proliferation of PRMT6 loss‐of‐function cells compared with control cells (**Figure** **7**A and Figure S4C, Supporting Information). Thus, Olaparib was selected for subsequent experiments. Olaparib treatment also increased the oscillation of PER3 expression, thereby regulating the uniform circadian clock by dissociating the PRMT6/PARP1/CRL4B complex. Additionally, PRMT6 gain‐of‐function restored the circadian rhythm disrupted by Olaparib (Figure 7B,C and Figure S4D,E, Table S3, Supporting Information). Meanwhile, in breast cancer cells treated with Olaparib, withdrawing PER3 returned PER3 oscillation to baseline (Figure 7D,E and Figure S4E, Table S3, Supporting Information). Although Olaparib exerts anticancer effects by inhibiting the DNA repair process, our phenotypic results indicate that it can also regulate tumor proliferation and metastasis by modulating circadian rhythms. In overexpressing PRMT6 cells, Olaparib treatment reduced the proliferation and invasive ability of breast cancer cells compared with the control group, whereas PER3 deletion rescued these effects (Figure 7F–H). This observation provides an innovative insight that PARP1 inhibitors exert anticancer effects by regulating circadian rhythm via a novel molecular mechanism.

Then, we sought to determine whether simultaneous targeting of the enzymatic activity of PRMT6 and PARP1 elicits synergetic effects against breast cancer tumorigenicity. To this end, cells were treated with PARP1 and PRMT6 inhibitors (Olaparib and EPZ020411). PAR and H3R2me2a expression were detected to represent the efficiency of PARP1 and PRMT6 enzymatic activity following treatment with Olaparib and EPZ020411. Optimal inhibition of PARP1 enzymatic activity was observed upon treatment with 1 × 10^−6^ to 3 × 10^−6^ m Olaparib (Figure S4F, Supporting Information). Moreover, 0 × 10^−9^ to 100 × 10^−9^ m EPZ020411 reduced the levels of H3R2me2a in a dose‐dependent manner (Figure S4F, Supporting Information). The expression of PRMT6/PARP1/CRL4B complex target genes was all augmented following treatment with Olaparib and EPZ020411 (Figure 7I and Figure S4G, Supporting Information). Additionally, at optimal concentrations, monotreatment and combined treatment with Olaparib and EPZ020411 induced notable effects on the proliferation and invasion of breast cancer cell lines. Meanwhile, co‐treatment with both inhibitors more effectively reduced breast cancer cell tumorigenicity and metastasis compared with either monotreatment (Figure 7J,K and Figure S4H, Supporting Information).

Next, to further investigate the in vivo effects of the PRMT6 and PARP1 inhibitors (Olaparib and EPZ020411), alone and in combination, we conducted NOD‐SCID mice from MDA‐MB‐231 cells (*n* = 8) to evaluate the growth of tumors. The results showed that tumor growth was more strongly inhibited following combined administration of PRMT6 and PARP1 inhibitors, compared with either monotreatment (Figure 7L,M and Figure S5A, Supporting Information), indicating a potent synergetic antitumor effect of the inhibitor in breast cancer. We performed a pathological examination of the heart, liver, lungs, spleen, and kidneys to assess the effects of Olaparib and EPZ020411 on the major organs. No significant differences were observed in the histological features of the organs following mono or combination treatment, compared with the control group (Figure S5B, Supporting Information), representing no significant side effects of Olaparib and EPZ020411 inside the body. Meanwhile, Ki‐67 staining further revealed that combination treatment inhibited tumor proliferation more strongly than either monotreatment (Figure 7O). To further validate whether Olaparib and EPZ020411 exhibit antitumor effects via regulation of the circadian clock in vivo, we assessed the abundance of PER3 via immunohistochemical (IHC) staining and western blotting. Compared with the control, Olaparib or/and EPZ020411 treatment significantly increased PER3 levels (Figure 7N,O and Figure S5C,D, Supporting Information). These data support the idea that the integrity of the PRMT6/PARP1/CRL4B complex allows it to bind to the promoters of target genes, however, loss of PRMT6 enzymatic activity prevents the transcriptional repression function of the complex. Hence, although the PRMT6 and PARP1 inhibitors exhibit unique effects, targeting PARP1 and PRMT6 exerts synergetic effects on breast cancer progression by targeting the circadian rhythm.

### 2.7 PRMT6 is Associated with Poor Clinical Outcomes as a Prospective Cancer Biomarker

**Figure 8:**
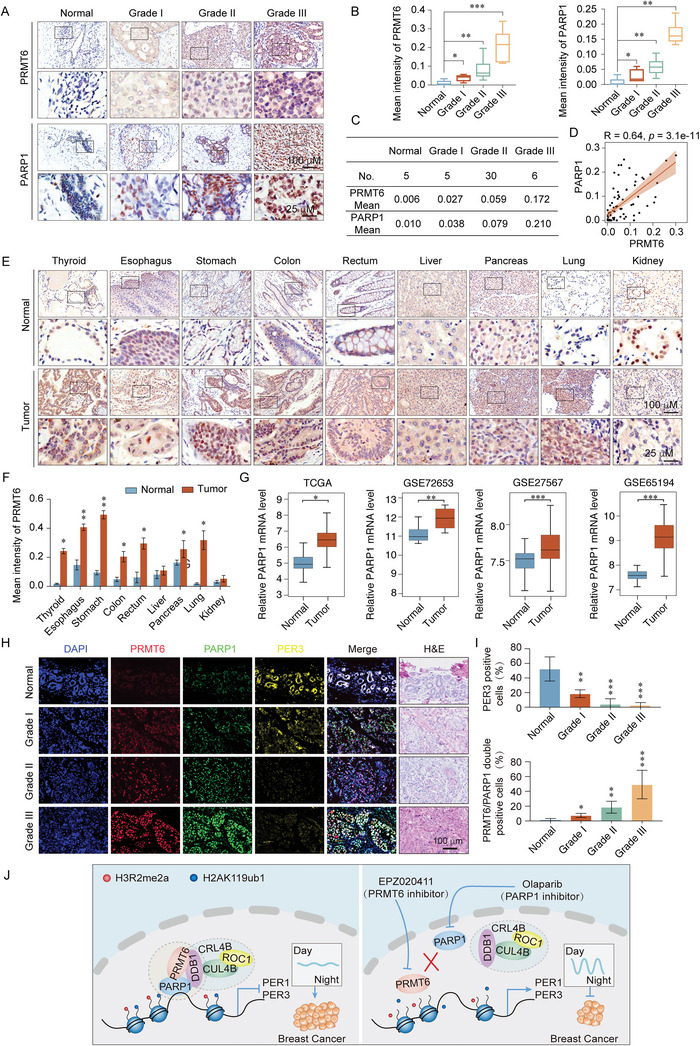
PRMT6 is associated with poor clinical outcomes and represents a prospective cancer biomarker. A) Immunohistochemical staining of PRMT6 and PARP1 in normal breast tissue and breast cancer tumors (histological grades I, II, and III). B,C) Quantification of PRMT6 and PARP1 immunostaining by means of intensity, as calculated using Image J. Total of 46 samples were analyzed. D) Relative level of PRMT6 plotted against that of PARP1. E,F) Immunohistochemical staining of PRMT6 in paired tumor tissues versus adjacent normal tissues. G) Analysis of public datasets (TCGA BRAC, GSE72653, GSE27567, and GSE65194) for *PARP1* mRNA expression in normal and breast cancer tissues. H) Representative images from immunofluorescence staining of PRMT6, PARP1, and PER3 in normal breast tissues and breast cancer tumors (histological grades I, II, and III). I) Proportion of PER3, PRMT6/PARP1 positive areas in normal breast tissue and breast cancer tumors (histological grades I, II, and III) analyzed using Image J. Total of 40 samples were analyzed. J) Graphic model as discussed in the text. The proposed regulatory mechanisms of the PRMT6/PARP1/CRL4B complex in regulating the circadian rhythm of breast carcinogenesis. B,F,G,I) Error bars represent the mean ± SD (*p < 0.05, **p < 0.01, ***p < 0.001). Two‐tailed unpaired *t*‐test.

To investigate the regulation of PRMT6 and PARP1 in breast cancer tissues, we assessed their protein levels in breast carcinoma samples of different histological grades, as well as in normal mammary tissues via IHC staining assays. PRMT6 and PARP1 expression were elevated in breast cancer samples and were positively correlated with histological grade (**Figure** **8**A–C). Thus, PRMT6 expression was positively correlated with that of PARP1 in breast cancer samples (Figure 8D). Analysis of online datasets further revealed augmented PRMT6, PARP1, CUL4B, and DDB1 levels in breast cancer tissues compared with normal tissues (Figure S5F, Supporting Information). Additionally, high levels of PRMT6 were represented in multiple cancer samples including thyroid, esophagus, stomach, colon, rectum, pancreas, and lung cancers, compared with paired normal tissues (Figure 8E,F). Based on TCGA and GEO datasets analysis, we found that PARP1 was significantly upregulated in breast cancer (Figure 8G).

We also validated the co‐expression of PARP1, PRMT6, and PER3 in normal breast tissue and three different grades breast cancer tissues using immunofluorescent staining (IF, Figure 8H). The co‐staining results indicated that PRMT6 and PARP1 abundances were elevated in breast cancer samples and were positively correlated with histological grades. Meanwhile, PER3 protein abundance was decreased, and was inversely correlated with the abundance of PRMT6 and PARP1, in breast cancer samples (Figure 8I). Elevated *PARP1*, or reduced *PER3* levels, were correlated with poor overall survival in breast cancer patients (Figure S5E, Supporting Information). The published datasets were analyzed to better understand the association between PRMT6, PARP1, DDB1, and PER3 in breast cancer. The level of *PRMT6* was found to be positively correlated with *PARP1* and *DDB1*, in contrast, the level of *PER3* was strongly and inversely associated with *PRMT6*, *PARP1*, and *DDB1* (Figure S5G, Supporting Information). Together, these data confirm that PRMT6 is an oncogene and represents a potential latent diagnostic marker for breast cancer.

## 3 Discussion

The current study results indicate that PRMT6 binds to PARP1, recruiting the CRL4B complex which, together, stimulate histone methylation and ubiquitination modification, thereby promoting breast cancer progression (Figure 8J). As such, the carcinogenic mechanism of the PRMT6/PARP1/CRL4B complex is a promising potential therapeutic target.

PRMT6, an integral methyltransferase, participates in many cellular regulatory events, including metabolism, senescence, DNA damage, differentiation, and proliferation.^[^ ^17^ ^]^ We found that PRMT6 is upregulated in seven solid tumor types, including breast, thyroid, esophagus, and colon tumors. PRMT6 participates in regulating DNA base excision repair by forming a complex with DNA polymerase *β* (Pol *β*), and methylating Pol *β* R83 and R152 to mitigate DNA strand breaks.^[^ ^2^ ^]^ PRMT6 is also a novel *TP53* transcriptional repressor that binds directly to the *TP53* promoter and catalyzes the repressive marker H3R2 asymmetric dimethylation, thereby preventing cellular senescence.^[^ ^18^ ^]^ As a transcriptional coactivator, PRMT6 can be recruited to the *NF‐*𝜅*B* promoter by interacting directly with RelA, affecting the histone coding and methylation of other chromatin‐associated proteins to promote transcription.^[^ ^19^ ^]^ Similarly, our transcriptomic analysis revealed that PRMT6‐knockdown tumor cells were enriched in DNA repair, senescence, and EMT‐related pathways and that PRMT6 participated in HR and NHEJ repair, regulating DNA damage and disrupting chromatin accessibility. Further, PRMT6 enhanced breast cancer cell proliferation, invasiveness, metastasis, and EMT, thus, promoting cancer progression. Hence, PRMT6 possesses transcriptional repression and transcriptional activation functions, thus, additional research is needed to elucidate the role and molecular mechanisms of PRMT6 in tumor progression, and to develop antitumor strategies.

During DNA repair, PARP1 interacts with target proteins or transcriptionally regulates target genes via PARylation. In breast cancer, it interacts directly with the CW‐ZF domain of MORC2, and PARylates MORC2 at the E516 and D517 residues. When DNA strands break, MORC2 PARylation occurs at the damaged sites, activating ATPases and chromatin remodeling for DNA repair.^[^ ^15^ ^]^ PARP1 also intracellularly recruits BRG1 and SIRT1 to DNA double‐strand break sites, promoting chromatin remodeling and HR‐dependent repair.^[^ ^20^ ^]^ In macrophages, PARP1 PARylates STAT1*α* to facilitate STAT1*α* phosphorylation to modulate enhancer activation, and regulate IFN *γ*‐dependent transcription and downstream proinflammatory responses.^[^ ^21^ ^]^ Meanwhile, we found that the PARP1 BRCT domain interacted directly with PRMT6 without PARylation modification. Similarly, PARP1 also interacted directly with the DDB1 protein in the CRL4B complex without PARylation modification. This implies that the PRMT6/PARP1/CRL4B complex is not post‐translationally modified and that PARP1 instead synergistically regulates downstream pathway transcription. Indeed, PARP1 plays multitudinous roles in transcriptional regulation, acting as a transcriptional activator or repressor in different physiological processes and disease states. More specifically, PARP1 functions as a transcriptional activator by localizing to RNA polymerase II‐transcribed promoters and preventing the localization of histone H1 to these promoters.^[^ ^22^ ^]^ Moreover, in breast cancer, PARP1 participates in maintaining the breast cancer epithelial cell phenotype by transcriptionally coactivating the transcription factor *GATA3*, thus, enhancing *CCND1* transcription in competition with histone H1 on chromatin.^[^ ^23^ ^]^ Our findings reveal that PRMT6 and PARP1 first assemble in the nucleus as a simple complex, which is then captured by DDB1 to bind the CRL4B complex, forming the PRMT6/PARP1/CRL4B transcriptional repressor complex. Thus, the presence or absence of the CRL4B complex does not affect PRMT6/PARP1 assembly, conversely, while PRMT6 or PARP1 knockdown causes dissociation of the PRMT6/PARP1/CRL4B complex, it does not affect the expression of each component. However, the assembly sequence of the PRMT6/PARP1/CRL4B complex might cause different physiological and pathological effects in other tissues, which requires further investigation.

The CRL4B complex comprises DDB1 as a linker, the small RING protein ROC1, and a cullin protein CUL4B. CUL4B does not typically bind directly to substrates but relies on substrates to recruit receptors, which are attached to the cullin complex by the linker protein DDB1.^[^ ^24^ ^]^ The CRL4B complex plays an important role in gene silencing, heterochromatin formation, and chromosome condensation via diverse mechanisms. Aberrant expression of CRL4B has been reported in a variety of diseases and is highly expressed in several solid tumors, where it promotes tumorigenesis by affecting tumor suppressors.^[^ ^25^ ^]^ Herein, the PRMT6/PARP1/CRL4B complex modified H3R2me2a and H2AK119ub leading to the disruption of circadian rhythms and transcriptional repression of key genes within the DNA repair pathway, including the tumor suppressor genes *PER1* and *PER3*. Interestingly, the presence of the CRL4B complex did not affect the occupancy of PRMT6/PARP1 at the target gene promoters. In contrast, PRMT6 and PARP1 are required for the occupancy of the CRL4B complex at these promoters. This is consistent with our previous conjecture regarding the assembly sequence of the PRMT6/PARP1/CRL4B complex. *PER1* and *PER3* are core clock genes in circadian rhythms and play decisive roles in the oscillatory gene expression loop. PER1 links circadian rhythms to the cell cycle by interacting with the checkpoint proteins ATM and CHK2 to reduce the levels of WEE1, Cyclin B1, and CDC2.^[^ ^26^ ^]^ PER1 inhibition disrupts the cell cycle and promotes cancer development. Meanwhile, the PER1/GPX1 complex augments GPX activity to improve mitochondrial oxidative phosphorylation activity and protects against mitochondrial surface‐imposed oxidative stress. Hence, PER1 deficiency contributes to a rhythmic dysregulation of daily oxygen consumption by impairing mitochondrial function.^[^ ^27^ ^]^ In oral squamous carcinoma, PER1 forms a complex with RACK1 and PI3K, affecting PI3K half‐life to impede glycolysis, glucose uptake, and cell proliferation.^[^ ^28^ ^]^ Moreover, examination of 203 colon cancer patients revealed PER3 dysregulation, identifying it as a promising biomarker for diagnosis and prognosis of colon cancer.^[^ ^29^ ^]^ Collectively, our current findings demonstrate that the PRMT6/PARP1/CRL4B transcriptional‐repression complex interferes with circadian rhythm.

Epidemiological investigations have revealed that circadian rhythm disruptions, such as chronic insomnia or sleep pattern reversal, are associated with high risk of various cancers. A 2006 study found that circadian rhythm disruption due to chronic jet lag both increases spontaneous hepatocellular carcinoma incidence and accelerate tumor progression. Metabolomic and transcriptomic analyses have further revealed the mechanism by which circadian rhythm disruption induces hepatocellular carcinoma. That is, chronic jet lag triggers dysregulation of carnitine transport and hastens cytoplasmic glycolysis for lipid synthesis and storage, culminating in sustained overall shifts in hepatic metabolism; such shifts also occur in most cancers.^[^ ^30^ ^]^ Similarly, in mice with lung cancer, clock gene mutations reduce survival and promote tumor progression. The clock genes that elevate c‐Myc expression contribute to cancer progression by promoting cell proliferation and metabolic dysregulation.^[^ ^31^ ^]^ Meanwhile, it is worth noting that in the current study, PRMT6/PARP1 knockdown obstructed breast cancer proliferation and metastasis by deregulating the transcriptional repression of *PER3*, however, *PER3* knockdown rescued the inhibition of cancer progression. Thus, the PRMT6/PARP1/CRL4B complex maintains carcinoma progression by reducing the amplitude of circadian rhythms and circadian oscillations. Additionally, we found that inhibition of PARP1 enzymatic activity by Olaparib disrupted assembly of the PRMT6/PARP1/CRL4B complex. Olaparib alters circadian clock oscillation by incrementing the amplitude of the clock gene *PER3* in breast cancer. As the first‐in‐class PARP inhibitor, Olaparib has been approved for the clinical treatment of advanced ovarian cancer, metastatic breast cancer, pancreatic cancer, and prostate cancer. Interestingly, inhibition of PRMT6 enzymatic activity did not damage the integrity of the PRMT6/PARP1/CRL4B complex, but prevents the transcriptional repression of clock genes by the complex. Our current findings reveal that the enzymatic activity of PRMT6 and PARP1 is crucial in different links of transcriptional repressions, thus, for breast cancer cells with upregulated *PRMT6* expression, administration of combined PRMT6 and PARP1 inhibitors may obstruct tumor progression by recovering the natural circadian rhythm.

## 4 Conclusion

Although the comprehensive mechanisms of the PRMT6/PARP1/CRL4B complex remain to be fully elucidated, our study reveals that the PRMT6/PARP1/CRL4B complex transcriptionally represses the core clock gene *PER3*, interrupting circadian clock oscillation, thereby promoting tumor progression. Hence, the PRMT6/PARP1/CRL4B complex might serve as an oncogenic target in breast cancers, and PRMT6 as a novel biomarker for PARP inhibitor monotherapy.

## 5 Experimental Section

### Antibodies and Reagents

The antibodies were listed as follows: anti‐FLAG (F1408), anti‐CUL4B (C9995), anti‐*β*‐actin (A1978), anti‐HDAC1 (H3284), anti‐HDAC2 (H3159), anti‐Vimentin (V6630) and anti‐Fibronectin (F3648) from Sigma‐Aldrich; anti‐DDB1 (sc‐25367) and anti‐PER1 (sc‐398890) from Santa Cruz Biotechnology; anti‐ROC1 (ab2977), anti‐H3 (ab1791), anti‐H4 (ab17840), anti‐H3R2me2a (ab194706), anti‐H3R2me2s (ab194684), anti‐H4R3me2s (ab5823), antiH4R3me2a (ab129231), anti‐H3R8me2s (ab130740), anti‐H3R8me2a (ab127163), anti‐H3K4me2 (ab32356), anti‐H3K36me1 (ab176920), anti‐H3K36me2 (ab176921), and anti‐H3K36me3 (ab194677) from Abcam; anti‐H2AK119ub1 (05‐678) from Millipore; anti‐*α*‐catenin (610193), anti‐*γ*‐catenin (610253), anti‐E‐cadherin (610181), and anti‐N‐cadherin (610920) from BD Bioscience; anti‐PRMT6 (14641S), anti‐PARP1 (9532T), and anti‐HER2 (4290) from Cell Signaling Technology; anti‐PER3 (12550‐1‐AP), anti‐LDHA (21799‐1‐AP), anti‐BHLHE40 (17895‐1‐AP), and anti‐CSNK1A1 (55192‐1‐AP) from Proteintech; anti‐PRMT6 (sc‐271744), anti‐PARP1 (sc‐8007), and anti‐PER3 (sc‐517227) from Santa; PARP inhibitor Olaparib (T3015) and Veliparib (T2591) and PRMT6 inhibitor EPZ020411 (T4334) from Topscience; small interfering RNAs targeting human PRMT6 (esiRNAs) (EHU125311) from Sigma‐Aldrich.

### Cell Culture and Transfection

The cell lines MDA‐MB‐231 and MCF‐7 were obtained from the American Type Culture Collection (ATCC, Manassas, VA, USA). Cells were cultured in Dulbecco's modified Eagle's medium (DMEM, Biological Industries, Beit‐Haemek, Israel) with 10% fetal bovine serum, 100 U mL^−1^ penicillin, and 100 U mL^−1^ streptomycin (Gibco BRL, Grand Island, NY, USA). For cell transfection experiments, MDA‐MB‐231 cells were transfected by Lipofectamine 2000 agent, and MCF‐7 cells were transfected by Turbofect agent (Invitrogen, Carlsbad, CA) according to the manufacturer's instructions. 10 µL Lipofectamine 2000 agent or 6 µL Turbofect agent in Optin‐MEM medium was diluted and diluted DNA was added to a diluted transfected agent and then incubated 15 min to add DNA–lipid complex to cells. Each experiment was performed in triplicate and repeated at least three times. The information for the plasmid used is listed in Table S4 in the Supporting Information.

### Lentiviral Production and Infection

Recombinant lentiviruses expressing GFP‐tagged shPRMT6, shPARP1 were constructed by Shanghai GenePharma Co., Ltd. (Shanghai, China). The lentiviral expressing GFP‐tagged PRMT6 was constructed by Shanghai GenePharma Co., Ltd. (Shanghai, China). Recombinant lentiviruses expressing Cherry‐tagged shPER3 were constructed by Shanghai GeneChem Co., Ltd. (Shanghai, China). The lentiviral expressing GFP‐tagged PARP1 was constructed by Shanghai GeneChem Co., Ltd. (Shanghai, China). Concentrated viruses were used to infect 5 × 105 cells in a 60 mm dish with 8 mg mL^−1^ polybrene. Infected cells underwent sorting for target expression. Stably expressing shPRMT6 and shPARP1 MDA‐MB‐231 and stably overexpression PRMT6 and PARP1 were screened by 2 µg mL^−1^ puromycin for 48 h. Stably expressing shPER3 MDA‐MB‐231 were screened by 40 µg mL^−1^ blasticidin for 10 days. The shRNA sequences used are listed in Table S5 in the Supporting Information.

### RNA‐Sequencing Analysis

Total RNA was extracted from shSCR MDA‐MB‐231 cells and shPRMT6 MDA‐MB‐231 cells using the TRIzol reagent (Roche, Basel, Switzerland). The total RNA was sent to the Beijing Genomics Institute (BGI, Shenzhen, China). Then the company sequenced six samples using DNBSEQ platform, averagely generating about 6.68G Gb bases per sample. The average mapping ratio with reference genome was 89.99%, and the average mapping ratio with gene was 70.57%; 15 758 genes were identified. Raw reads were mapped to the human reference genome (hg19). The transcriptome was analyzed by the TopHat2 package. Htseq‐count v0.6.0 was used to quantize transcript abundances. All RNA‐seq data are available on https://www.ncbi.nlm.nih.gov/geo/query/acc.cgi?acc=GSE210948.

### Real‐Time Quantitative Polymerase Chain Reaction Analysis

Total RNA was extracted from shSCR, shPRMT6, shPARP1, shCUL4B, and shDDB1 MDA‐MB‐231 cells using the TRIzol reagent (Roche, Basel, Switzerland) to perform reverse transcription using the RevertAid First Strand cDNA Synthesis Kit (Roche, Basel, Switzerland). The primers used for the detection are shown in Table S6 in the Supporting Information. *β*‐actin was used as an internal reference.

### Mouse Xenograft Models

All animal treatment and procedures were approved by the Capital Medical University Institutional Animal Care (No. 110011211113264741). shSCR, shPRMT6, shPER3, Vector, PRMT6 MDA‐MB‐231 (5 × 10^6^ cells) were inoculated into the left abdominal mammary fat pad of 6 weeks old female NOD‐SCID mice to assess tumor growth, respectively. For drug efficacy experiment, 32 mice were divided into control, Olaparib, EPZ020411, and combination‐treated group. When the tumor size was ≈100 mm^3^, the treatment was initiated with Olaparib (i.p, 50 mg kg^−1^, every other day for 4 weeks) or EPZ020411 (i.p, 10 mg kg^−1^, every other day for 4 weeks). After 4 weeks of treatment, all mice were sacrificed according to ethical guidelines. Tumors were excised and analyzed. Tumor volume was measured using a Vernier caliper and calculated by the following formula: *π*/6 × length × width^2^.

### Immunoprecipitation and Western Blot

For immunoprecipitation assays, the cells were washed and collected with cold phosphate‐buffered saline (PBS) after the cells were full grown on the 10 cm dish to lyse the cells with cell lysis buffer for 30 min at 4 °C. Lysed cells were centrifuged with 12 000 × *g* for 10 min, then supernatant was aspirated to added antibodies or normal rabbit/mouse immunoglobin G (IgG) for incubating overnight. The protein A/G Sepharose beads were added for further incubation for 2 h on the next day. Then the beads were washed and proteins were denatured to be subjected to 10% sodium dodecyl‐sulfate polyacrylamide gel electrophoresis (SDS‐PAGE). Immunodetection was performed by enhanced chemiluminescence (ECL System, The rmo Fisher Scientific, Waltham, MA, USA).

### MNase Digestion Assay

1 × 10^6^ cells were collected and suspended in lysis buffer for 5 min, then centrifuged at 2000 × *g* for 5 min to collect the nuclear pellets. The sediment was suspended with 50 µL glycerol buffer and mixed with an equal volume of MNase buffer to incubate for 5 min at 37 °C. The reaction was stopped with a final concentration of 10 × 10^−3^ m ethylenediaminetetraacetic acid. 3 µL of 25 mg mL^−1^ proteinase K and 20 µL of 10% SDS were added. The samples were incubated overnight at 37 or 50 °C. Genomic DNA was purified for electrophoretic separation.

### Comet Assay

Alkaline comet assays were performed with shSCR, shPRMT6, Vector, PRMT6 MDA‐MB‐231 cells following the treatment of DOX to induce DNA damage using a Comet Assay kit according to the manufacturer's instruction. Briefly, preparation of the agarose/cells slides to immerse slides successively into lysis solution and alkaline unwinding solution (pH > 13) for electrophoretic separation. DNA was visualized using 4′,6‐diamidino‐2‐phenylindole.

### HR and NHEJ Reporter Assay

DR‐GFP U2OS and EJ5‐U2OS cells were used to detect HR and NHEJ efficiency by transfecting I‐SceI plasmid and PRMT6 plasmid or siRNA PRMT6 to cause the restoration of GFP gene through HR or NHEJ repair. The percentage of GFP‐positive cells was calculated by fluorescence activated cell sorter (FACS) analysis.

### EdU Incorporation Assay

shSCR, shPRMT6, Vector, PRMT6 MDA‐MB‐231, and MCF‐7 cells were trypsinized and seeded into 96‐well plates at a density of 10^5^ mL^−1^ cells culturing overnight. Then, cells in the normal growth phase are examined for proliferation using an EdU assay kit according to the manufacturer's instructions (RiboBio, Guangzhou, China).

### Transwell Assay

For transwell invasion assay, shSCR, shPRMT6, Vector, PRMT6 MDA‐MB‐231, and MCF‐7 cells were seeded with serum‐free media in the upper chamber of the transwell dusted with diluted Matrigel. For transwell migration assay, cells were seeded with serum‐free media in the upper chamber of the transwell. The chamber was transferred to a 24‐well containing 10% fetal bovine serum. The MDA‐MB‐231 cells were incubated for 20 h and MCF‐7 cells for 48 h at 37 °C. After incubation, cells were fixed, washed, and stained with crystal violet for half an hour, then image was captured using a light microscope.

### Colony Formation Assay

The colony‐formation ability of shSCR, shPRMT6, Vector, PRMT6 MDA‐MB‐231, and MCF‐7 cells was measured by the clonogenic assay. MDA‐MB‐231 and MCF‐7 were seeded into 6‐well plates. The MDA‐MB‐231 cells were cultured for 10 days (2000 cells per well) and MCF‐7 cells for 15 days (5000 cells per well). Then, cells were fixed, washed, and stained with crystal violet for half an hour, then image was captured using a light microscope.

### Immunopurification and Mass Spectrometry

HEK293T cells stably expressing FLAG‐PRMT6 or FLAG‐PARP1 were generated by transfecting the cells with FLAG‐tagged PRMT6 or FLAG‐tagged PARP1 plasmids. Cell lysate was obtained from ≈5 × 10^8^ cells and applied to the FLAG column to elute the FLAG protein complex according to the manufacturer's instructions. Fractions of the bed volume were collected and undertaken on SDS‐PAGE precast gels, silver stained, and subjected to liquid chromatography‐tandem mass spectrometry sequencing and data analysis.

### Glutathione S‐Transferase (GST) Pull‐Down Experiments

GST fusion protein was constructed and purified with glutathione‐Sepharose 4B beads, meanwhile in vitro transcription and translation experiments were performed with rabbit reticulocyte lysate (TNT Systems; Promega, Madison, WI, USA). For GST pull‐down assays, the GST fusion protein and the in vitro translated products were mixed with incubated in binding buffer. The beads were washed and resuspended in SDS‐PAGE loading buffer and further western blotting was performed.

### CUT&Tag Sequencing

CUT&Tag is an innovative technique that replaces the traditional ChIP‐Seq for studying DNA‐protein interactions. Preparation of ≈10^4^ cells was for each CUT&Tag assay to perform the following steps using NovoNGS CUT&Tag 2.0 high‐sensitivity kit (Novoprotein, Shanghai, China): absorbing live cells by magnetic beads; increasing cell membrane permeability with digitonin; incubating with PRMT6 or PARP1 primary antibodies and the secondary antibody; cutting the target region with Mg^2+^ activated the Tn5 enzyme activity; adding connectors to both ends of the cleaved fragment, and finally the complete sequencing library was obtained by PCR amplification. In‐depth whole genome DNA sequencing was performed by the CapitalBio Corporation, Beijing. Raw sequencing image data were examined using the Illumina HiSeq 2000 and further analyzed by MACS. The ChIP peak annotation, comparison, and visualization of PARP1 and PRMT6 were analyzed by IGV, an R package for ChIP peak annotation, comparison, and visualization. All ChIP‐seq data are available on https://www.ncbi.nlm.nih.gov/geo/query/acc.cgi?acc = GSE210947.

### Chromatin Immunoprecipitation (ChIP) and Re‐ChIP

ChIP and Re‐ChIPs were performed in MDA‐MB‐231 cells as previously described.^[^ ^32^ ^]^ Briefly, for ChIP assay, 1 × 10^7^ cells were crosslinked, sonicated, precleared, and incubated with indicated antibody, then DNA was extracted and precipitated. For Re‐ChIP assays, immune complexes were eluted from the beads to dilute by ChIP dilution buffer and subjected to a second immunoprecipitation reaction. DNA template enrichment was analyzed by PCR or qPCR using primers for each target gene promoter. The primer sequences are shown in Table S7 in the Supporting Information.

### Time‐Series Protein Assay

Time‐series protein assay in MDA‐MB‐231 cells was performed as described previously.^[^ ^33^ ^]^ MDA‐MB‐231 cells were seeded at 60 mm dishes and replaced with serum‐free DMEM after culturing for 48 h or 24 h. Then cells were treated with 200 nmol L^−1^ dexamethasone at 37 °C for 2 h, collected at a 4 h interval from 24 to 48 h.

### Tissue Specimens and Immunostaining Assays

Tissue Specimens were purchased from Shanghai Outdo Biotech Company (Shanghai, China). All human tissues were collected using protocols approved by the Ethics Committee of Cancer Hospital Chinese Academy of Medical Sciences, and informed consent was obtained from all patients. The assigned project number was NCC2021C‐179 in the experimental section of Figure 8. Breast, thyroid, esophagus, stomach, colon, rectum, liver, pancreas, lung, and kidney tumors and their corresponding normal tissues were rapidly fixed in 4% paraformaldehyde (Sigma‐Aldrich) at 4 °C overnight and embedded in paraffin for either immunohistochemistry staining or multiplex immunofluorescence according to a standard protocol. Sections were stained via incubation with 3,3'‐diaminobenzidine. IF staining was conducted by using a multiplex IF staining assay kit (Panovue, Beijing, China) according to the manufacturer's instruction.

### Statistical Analysis

Statistical analyses of all data were performed using GraphPad Prism (version 8.0, Graph Pad Software Inc., San Diego, CA, USA). All data were analyzed as the means ± SD by student's *t*‐test, two‐tailed unpaired *t* test, or two‐way analysis of variance (ANOVA) from at least three independent experiments unless otherwise noted. *p*‐values < 0.05 were considered statistically significant, with *p < 0.05, **p < 0.01, ***p < 0.001, respectively. Correlation analysis was performed using the Pearson correlation test. Breast tumor datasets were downloaded from http://www.ncbi.nlm.nih.gov/geo and the GSE numbers are shown in Figures 1A, 8G, and Figure S5C in the Supporting Information. Kaplan–Meier survival analysis in Figure 1B and Figure S5A in the Supporting Information was obtained from http://kmplot.com/analysis, log‐rank tests were used for the statistical analysis.

## Conflict of Interest

The authors declare no conflict of interest.

## Author Contributions

Y.W. conceived this project. T.Y., W.H., T.M., X.Y., J.Z., M.H., T.H., T.G., W.L., and D.Z. mainly conducted experiments. T.Y., H.Y., X.T., H.Q., Y.Y., and B.Y. acquired data. T.Y., W.H., and X.Y. analyzed data. T.Y., W.H., and Y.W. wrote the manuscript.

## Supporting information

Supporting Information

## Data Availability

All data needed to evaluate the conclusions in the paper are present in the paper and/or the Supplementary Materials. Additional data related to this paper may be requested from the authors.
