# Supplementary material for: The PRMT6/PARP1/CRL4B Complex Regulates the Circadian Clock and Promotes Breast Tumorigenesis
Source: Adv Sci (Weinh). 2023 Mar 20;10(14):2202737. doi: 10.1002/advs.202202737 (PMC10190619; doi:10.1002/advs.202202737)
Supplement: Supplementary file 1 — Supporting Information [file ADVS-10-2202737-s001.pdf]

ADVANCED SCIENCE
Open Access

Supporting Information

for *Adv. Sci.*, DOI 10.1002/advs.202202737

The PRMT6/PARP1/CRL4B Complex Regulates the Circadian Clock and Promotes Breast Tumorigenesis

*Tianshu Yang, Wei Huang, Tianyu Ma, Xin Yin, Jingyao Zhang, Miaomiao Huo, Ting Hu, Tianyang Gao, Wei Liu, Die Zhang, Hefen Yu, Xu Teng, Min Zhang, Hao Qin, Yunkai Yang, Baowen Yuan and Yan Wang**

Supporting Information

The PRMT6/PARP1/CRL4B complex regulates circadian clock and promotes breast tumorigenesis

*Tianshu Yang, Wei Huang, Tianyu Ma, Xin Yin, Jingyao Zhang, Miaomiao Huo, Ting Hu, Tianyang Gao, Wei Liu, Die Zhang, Hefen Yu, Xu Teng, Hao Qin, Yunkai Yang, Baowen Yuan, and Yan Wang**

Supporting Figures

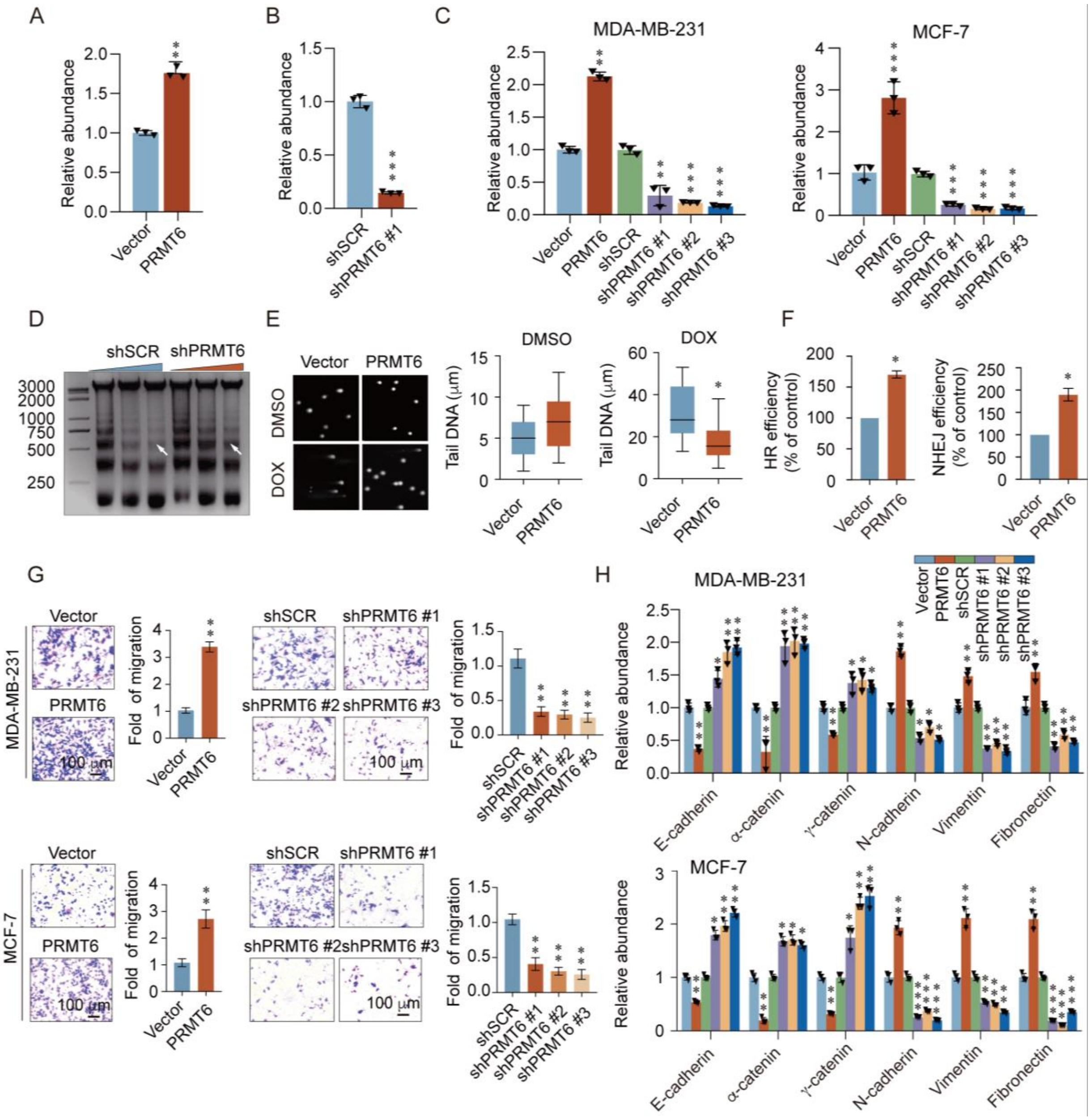

Figure S1. PRMT6 participate in breast cancer progression. (A) Quantitative protein expression by gray scanning of Figure 1H. (B) Quantitative protein expression by gray scanning of Figure 1I. (C) Quantitative protein expression by gray scanning of Figure 2A. (D) MNase digestion assay was performed in the stably PRMT6 KD (shPRMT6#1) and control MDA-MB-231 cells. (E) Overexpressed of PRMT6 MDA-MB-231 cells treated with DOX or DMSO for 2 h. DNA damage in these cells was measured using an alkaline comet assay (25 cells), and tail DNA are shown. (F) Homologous recombination (HR) efficiency, determined by FACS, for

PRMT6-overexpression DR-GFP U2OS cells (left panel). Non-homologous end joining (NHEJ) efficiency, determined by FACS, for PRMT6- overexpression EJ5-U2OS cells (right panel). (G) Transwell migration assay in the stable PRMT6 overexpression and PRMT6 KD (shPRMT6 #1, shPRMT6 #2, and shPRMT6 #3) MDA-MB-231 and MCF-7 cells. (H) Quantitative protein expression by gray scanning of Figure 2I. (A, B, C and H) Error bars represent the mean ± SD of three independent experiments (*$p < 0.05$, ** $p < 0.01$, *** $p < 0.001$). Two-tailed unpaired *t* test. (F and G) Bars represent the mean ± SD of three independent experiments (*$p < 0.05$, ** $p < 0.01$, *** $p < 0.001$). Student's *t*-test. shSCR, control scrambled shRNA.

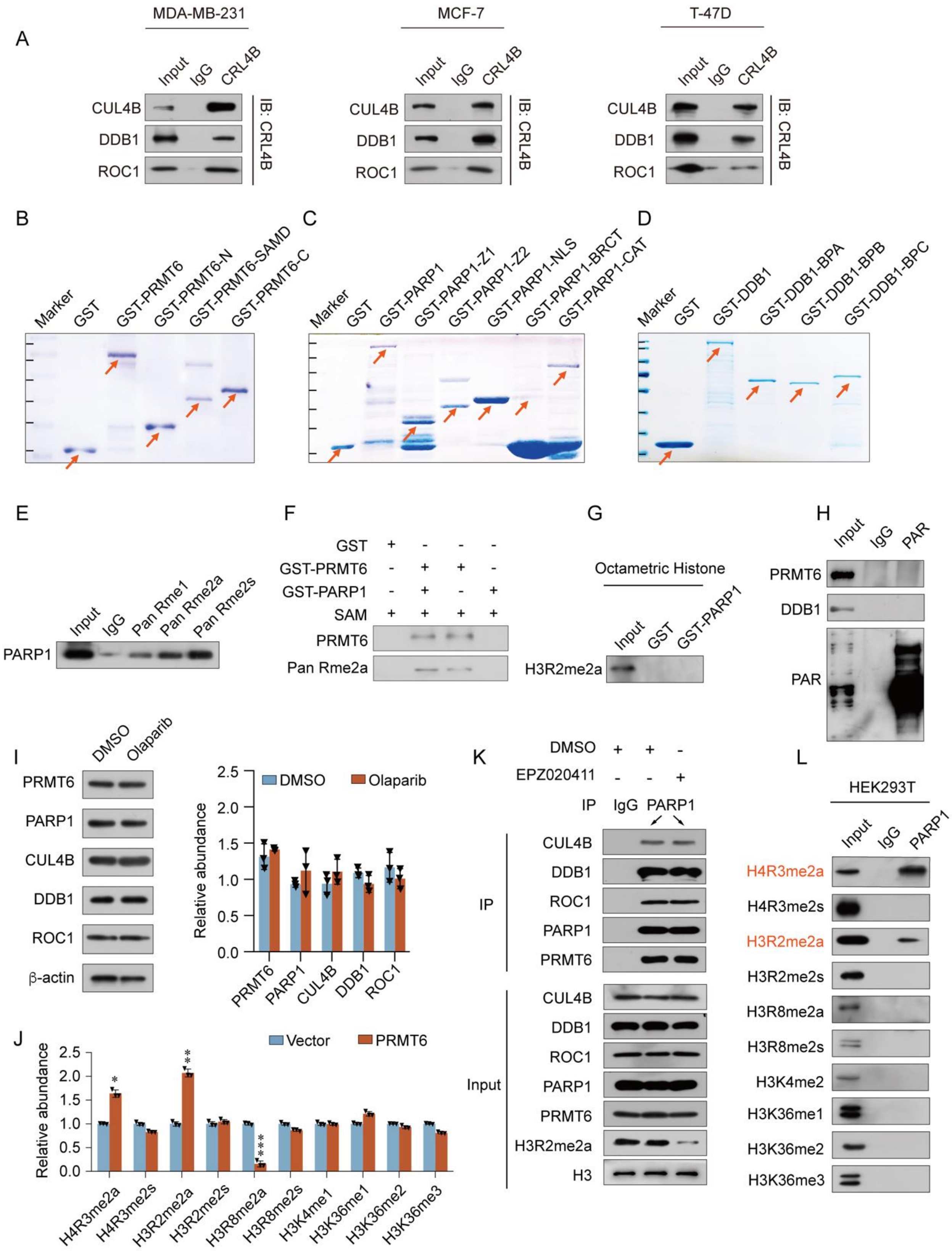

A
MDA-MB-231
MCF-7
T-47D
Input
IgG
CRL4B
CUL4B
DDB1
ROC1
IB: CRL4B
B
Marker
GST
GST-PRMT6
GST-PRMT6-N
GST-PRMT6-SAMD
GST-PRMT6-C
C
GST-PARP1
GST-PARP1-Z1
GST-PARP1-Z2
GST-PARP1-NLS
GST-PARP1-BRCT
GST-PARP1-CAT
D
GST-DDB1
GST-DDB1-BPA
GST-DDB1-BPB
GST-DDB1-BPC
E
Pan Rme1
Pan Rme2a
Pan Rme2s
PARP1
F
GST
GST-PRMT6
GST-PARP1
SAM
PRMT6
Pan Rme2a
G
Octametric Histone
H3R2me2a
H
PAR
PRMT6
DDB1
I
DMSO
Olaparib
PRMT6
PARP1
CUL4B
DDB1
ROC1
β-actin
Relative abundance
J
Vector
PRMT6
H4R3me2a
H4R3me2s
H3R2me2a
H3R2me2s
H3R8me2a
H3R8me2s
H3K4me1
H3K36me1
H3K36me2
H3K36me3
K
DMSO
EPZ020411
IP
IgG
PARP1
Input
H3
L
HEK293T
H3K4me2

Figure S2. PRMT6 and PARP1 do not post-translationally modify each other. (A) Association of PRMT6 with PARP1 and CRL4B complex in MDA-MB-231, MCF-7 and T-47D cells. Whole-cell lysates were prepared, and Co-IP was performed. (B, C and D) GST fused proteins purified from BL21 Escherichia coli. used in Fig. 3F, 3G and 3H. (E and F) PARP1 arginine methylation detected by immunoprecipitation and an in vitro methylation assay. MDA-MB-231 cells were immunoprecipitated with arginine panmonomethylated and pan-dimethylated antibody followed by immunoblotting with PARP1 antibody (upper panel). In vitro-purified PRMT6 was co-incubated with GST-fused PARP1, and the reaction mixes were immunoblotted with arginine pan-dimethylation asymmetry antibodies (lower panel). (G) GST pull-down experiments with bacterially expressed GST-PARP1 and histone octamers. Immunoblot using antibodies against H3R2me2a. (H) Lysates from MDA-MB-231 cells were immunoprecipitated with control IgG or an anti-PAR antibody, followed by immunoblotting with the indicated antibodies. (I) Western blot analysis of the indicated proteins in the MDA-MB-231 cells with Olaparib or DMSO treatment. (J) Quantitative protein expression by gray scanning of Figure 4I. (K) Lysates from MDA-MB-231 cells with EPZ020411 treatment were immunoprecipitated with control IgG or an anti-PARP1 antibody, followed by immunoblotting with the indicated antibodies. (L) Lysates from HEK293T cells were immunoprecipitated with control IgG or an anti-PARP1 antibody, followed by immunoblotting with the indicated antibodies. (I and J) Error bars represent the mean ± SD of three independent experiments (*$p < 0.05$, ** $p < 0.01$, *** $p < 0.001$). Two-tailed unpaired *t* test.

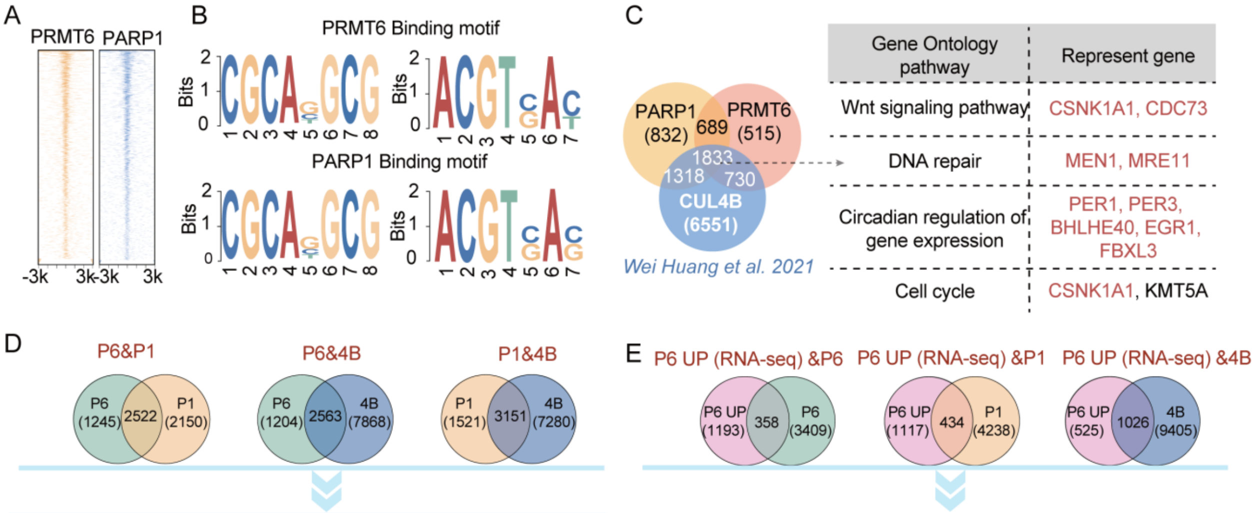

	P6&P1	P6&4B	P1&4B
Wnt signaling pathway	23	22	34
DNA repair	49	52	60
Circadian rhythm	12	12	13
Cell cycle	33	29	52
Hedgehog signaling pathway	11	12	12
Hippo signaling pathway	29	30	37
p53 signaling pathway	19	16	30
Cellular senescence	27	29	49
Tight junction	28	29	39
mTOR signaling pathway	25	21	36

	P6 UP&P6	P6 UP&P1	P6 UP&4B
Metabolic pathways	27	31	74
Cell cycle	4	7	9
p53 signaling pathway	3	4	6
Cellular senescence	3	5	11
Tight junction	4	4	13
mTOR signaling pathway	6	7	14
Estrogen signaling pathway	3	4	6
Circadian rhythm	2	2	2
Wnt signaling pathway	3	4	9
DNA repair	8	8	17

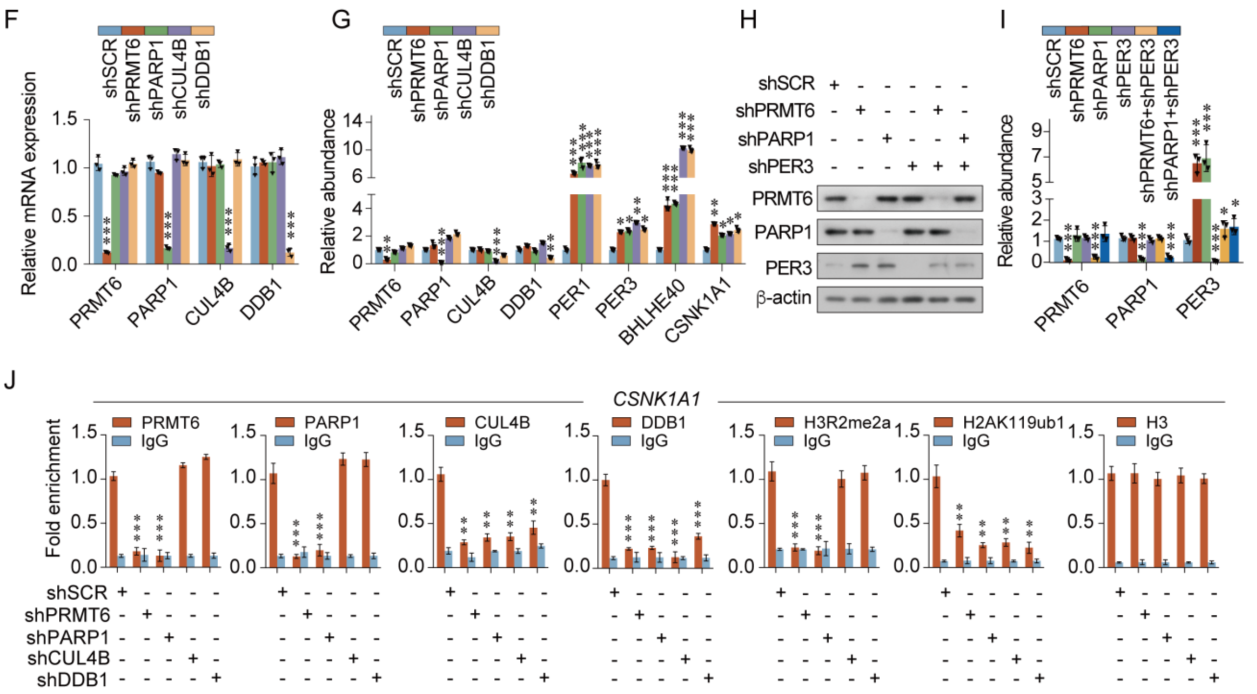

Figure S3. Identification of genome-wide transcription targets of the PRMT6/PARP1/CRL4B complex. (A) Heatmaps of PRMT6 and PARP1 ChIP-seq signals. (B) PRMT6 and PARP1 bound motifs were analyzed using MEME suite. (C) Venn diagram plots of promoters bound for PRMT6, PARP1 and CUL4B (left panel). A list of the 4 enriched GO pathways with representative genes comprising the 1833 overlapping target genes of PRMT6, PARP1 and CUL4B are also shown (right panel). (D) Venn diagrams of overlapped promoters bound by the PRMT6/PARP1 complex, the PRMT6/CRL4B complex and the PARP1/CRL4B complex; overlapping signaling pathways were functionally clustered using KOBAS. (E) Venn diagrams of overlapped RAN-seq and ChIP-seq results by PRMT6 UP/PRMT6, PRMT6 UP/PARP1 Complex and PRMT6 UP/CRL4B complex; overlapping signaling pathways were functionally clustered using KOBAS. P1, PARP1; P6, PRMT6, 4B, CUL4B. (F) Establishment of the stably PRMT6 KD, PARP1 KD, CUL4B KD, DDB1 KD, and control MDA-MB-231cells. qRT-PCR analysis of indicated mRNA levels in these cells. (G) Quantitative protein expression by gray scanning of Figure 5E. (H and I) MDA-MB-231 cells were infected with control shRNA (shSCR) or shRNAs targeting PRMT6, PARP1 and/or PER3 for the measurement of the indicated proteins by Western blotting. (J) qChIP analysis of the recruitment of indicated proteins on *CSNK1A1* promoters in MDA-MB-231 cells after transfection with control shRNA (shSCR) or shRNAs targeting PRMT6, PARP1, CUL4B or DDB1. Purified rabbit IgG was used as a negative control. (F and J) Bars represent the mean ± SD of three independent experiments (*$p < 0.05$, ** $p < 0.01$, *** $p < 0.001$). Student's *t*-test. (G and I) Error bars represent the mean ± SD of three independent experiments (*$p < 0.05$, ** $p < 0.01$, *** $p < 0.001$). Two-tailed unpaired *t* test.

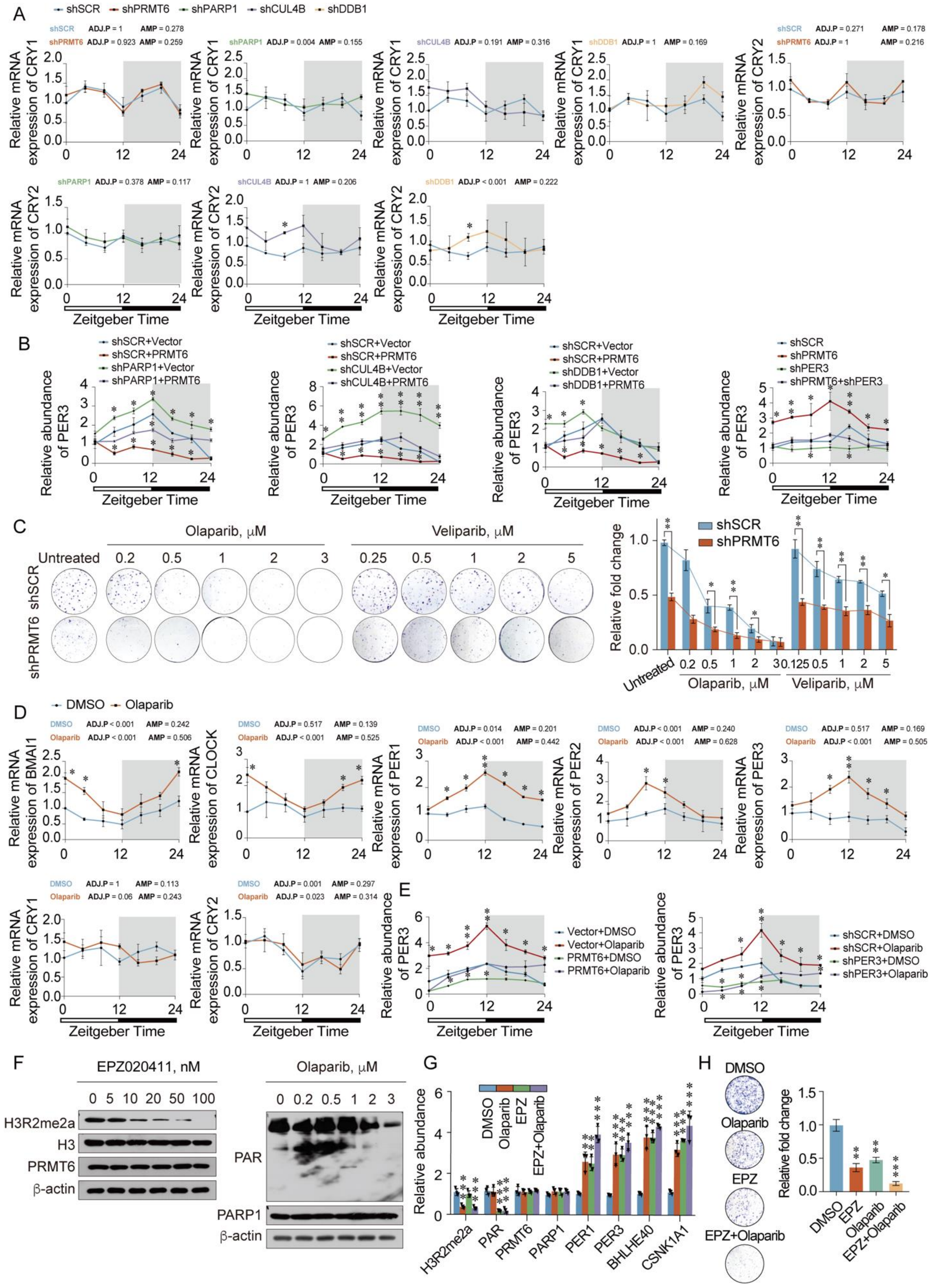

A
shSCR shPRMT6 shPARP1 shCUL4B shDDB1
Relative mRNA expression of CRY1
Relative mRNA expression of CRY2
Zeitgeber Time
B
Relative abundance of PER3
shSCR+Vector
shSCR+PRMT6
shPARP1+Vector
shPARP1+PRMT6
shCUL4B+Vector
shCUL4B+PRMT6
shDDB1+Vector
shDDB1+PRMT6
shPER3
shPRMT6+shPER3
C
Olaparib, μM
Veliparib, μM
Untreated
Relative fold change
D
DMSO Olaparib
Relative mRNA expression of BMAL1
Relative mRNA expression of CLOCK
Relative mRNA expression of PER1
Relative mRNA expression of PER2
Relative mRNA expression of PER3
E
Vector+DMSO
Vector+Olaparib
PRMT6+DMSO
PRMT6+Olaparib
shSCR+DMSO
shSCR+Olaparib
shPER3+DMSO
shPER3+Olaparib
F
EPZ020411, nM
H3R2me2a
H3
PRMT6
β-actin
PAR
PARP1
G
Relative abundance
EPZ
EPZ+Olaparib
H3R2me2a PAR PRMT6 PARP1 PER1 PER3 BHLHE40 CSNK1A1
H
DMSO
Olaparib
EPZ
EPZ+Olaparib

Figure S4. Formation of the PRMT6/PARP1/CRL4B complex functionally interrupts tumor-autonomous circadian clock. (A) qRT-PCR analysis of CRY1 and CRY2 in control, PRMT6 knockdown (KD), PARP1 KD, CUL4B KD, and DDB1 KD MDA-MB-231 cells. Bars represent the mean ± SD of three independent experiments (*$p < 0.05$, versus shSCR cells at 0 h). Two-way ANOVA. (B) Quantification of PER3 protein levels of Figure 6D, 6F, 6H and 6J. Bars represent the mean ± SD of three independent experiments (*$p < 0.05$, ** $p < 0.01$, versus control group at 0 h). Two-way ANOVA. (C) MCF-7 cells were infected with control shRNA (shSCR) or shRNAs targeting PRMT6 and treated with different doses of Olaparib or Veliparib. Representative images (left) and quantification of colony formation assay (right) from biological triplicate experiments are shown. (D) qRT-PCR analysis of BMAI1, CLOCK, PER2, PER2, PER3, CRY1, and CRY2 in DMSO- and olaparib-treated MDA-MB-231 cells. Bars: mean ± SD of three independent experiments (*$p < 0.05$, ** $p < 0.01$, versus DMSO cells at 0 h). Two-way ANOVA. (E) Quantification of PER3 protein levels of Figure 7C and 7E. Bars represent the mean ± SD of three independent experiments (*$p < 0.05$, ** $p < 0.01$, versus control group at 0 h). Two-way ANOVA. (F) Western blotting analysis using MDA-MB-231 cells treated with different concentrations of Olaparib and EPZ020411 with antibodies against the indicated proteins. (G) Quantitative protein expression by gray scanning of Figure 7I. EPZ, EPZ020411. (H) Colony formation assays of MDA-MB-231 cells treated with Olaparib and/or EPZ020411. EPZ, EPZ020411. (C, G and H) Bars represent the mean ± SD of three independent experiments (*$p < 0.05$, ** $p < 0.01$). Student's *t*-test.

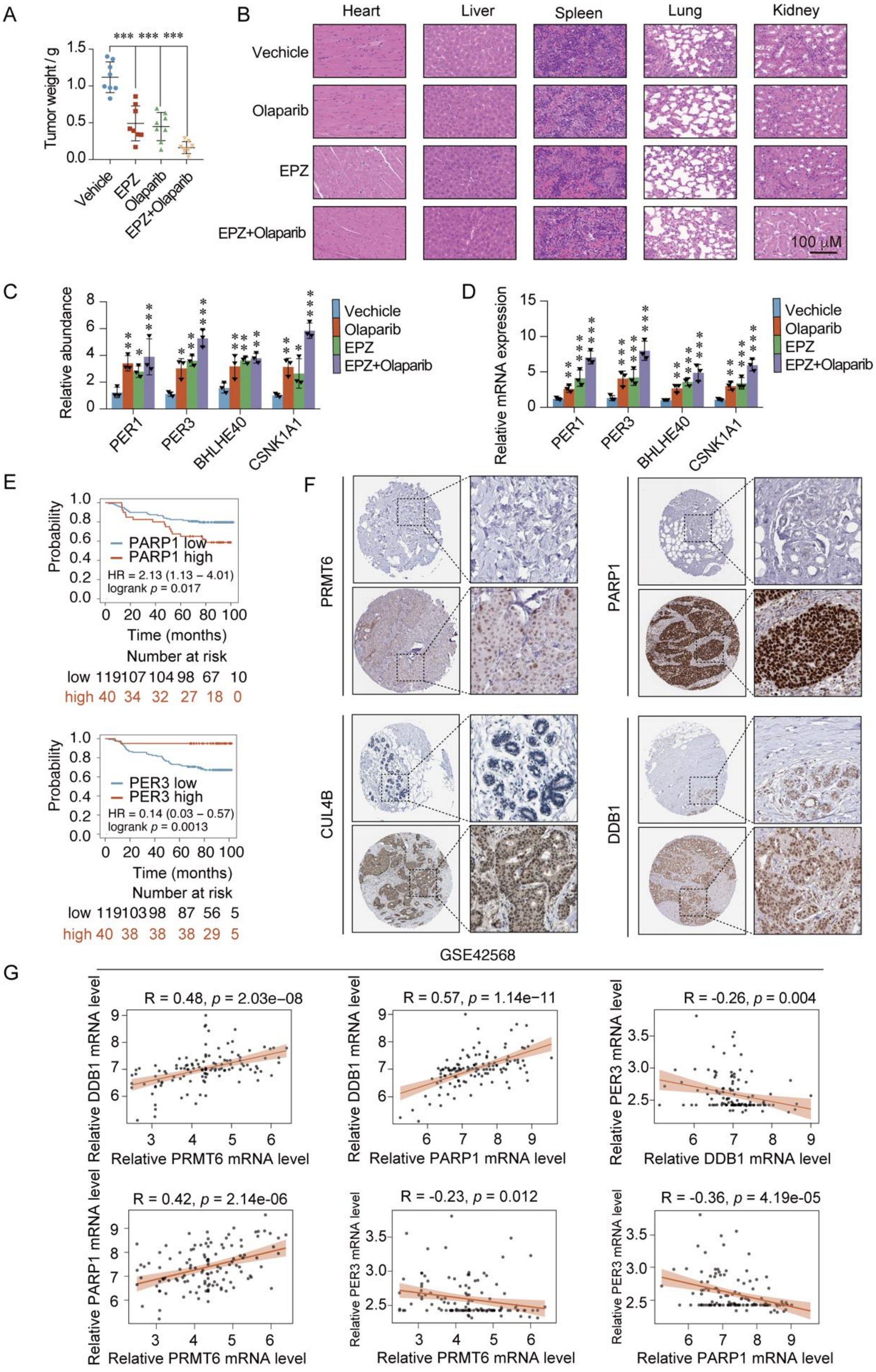
A
B
C
D
E
F
G
Tumor weight / g
Vehicle
EPZ
Olaparib
EPZ+Olaparib
Heart
Liver
Spleen
Lung
Kidney
Vechicle
Olaparib
EPZ
EPZ+Olaparib
100 μM
Relative abundance
Relative mRNA expression
PER1
PER3
BHLHE40
CSNK1A1
Probability
PARP1 low
PARP1 high
HR = 2.13 (1.13 − 4.01)
logrank p = 0.017
Time (months)
Number at risk
low 119107 104 98 67 10
high 40 34 32 27 18 0
PER3 low
PER3 high
HR = 0.14 (0.03 − 0.57)
logrank p = 0.0013
low 119103 98 87 56 5
high 40 38 38 38 29 5
PRMT6
PARP1
CUL4B
DDB1
GSE42568
R = 0.48, p = 2.03e−08
R = 0.57, p = 1.14e−11
R = -0.26, p = 0.004
R = 0.42, p = 2.14e-06
R = -0.23, p = 0.012
R = -0.36, p = 4.19e-05
Relative DDB1 mRNA level
Relative PRMT6 mRNA level
Relative PARP1 mRNA level
Relative PER3 mRNA level
Relative DDB1 mRNA level

Figure S5. The PRMT6/PARP1/CRL4B complex is associated with poor clinical outcomes as a prospective cancer biomarker.

(A) Tumors were harvested and weighed when the mice were sacrificed. Error bars represent mean ± SEM, ***$p < 0.001$. One-way ANOVA. (B) H&E staining of the heart, liver, spleen, lung, and kidney from the vehicle, EPZ, Olaparib and the combination groups. Scale bars, 100 μm. EPZ, EPZ020411. (C) Quantitative protein expression by gray scanning of Figure 7N. EPZ, EPZ020411. (D) qRT-PCR analysis of indicated mRNA levels in tumor tissues. (E) Kaplan–Meier survival analysis of the relationship between survival time and *PARP1* and *PER3* signature in breast cancer using the online tool. (F) Immunohistochemistry of the PRMT6, PARP1, CUL4B and DDB1 proteins in breast cancer and normal breast tissues from the Human Protein Atlas. (G) Analysis of published clinical datasets (GSE42568) for the expression of *PARP1*, *DDB1*, *PER3*, and expression of *PRMT6* by two-tailed unpaired *t*-test.

Supporting Tables

Table S1. Mass Spectrometry Analysis of PRMT6-containing Protein Complex.

Gene Symbol	Score	Coverage (%)	Peptides
PARP1	69.13	40.14	84
CUL4B	4.7	6.222	3
DDB1	22.96	14.65	15
HDAC1	4.14	6.846	2
HDAC2	2.04	4.918	1
LDHA	3.64	14.16	2
FASN	98	26.9	91
DDX3X	47.42	46.07	82
DDX5	36.58	37.299	45
USP7	41.88	24.95	32
DHX9	41.85	22.05	31
VCP	23.95	23.95	27
TRIM28	23.43	33.41	25
ILF2	16.15	30.10	21
TCP1	21.04	34.88	21
PRRC2A	34.15	13.49	20
DDX41	34.03	29.37	20
IRS4	24.02	17.41	19
UBC	7.99	86.22	14
UBB	7.99	76.34	14
XRCC1	17.38	25.45	12
LARP1	15.42	18.07	12
PRMT5	15.28	19.56	11
UBBP4	3.06	36.72	11
ZGPAT	20.19	30.12	11
MAGED2	19.46	25.67	11
UBA1	18	12.38	11
MCM7	17.62	22.66	11

Table S2. Mass Spectrometry Analysis of PARP1-containing Protein Complex.

Gene Symbol	Score	Coverage (%)	Peptides
PRMT6	7.91	21.6	12
CUL4B	6.51	10.99	4
DDB1	4.43	7.50	3
HDAC1	4.19	9.75	2
HDAC2	3.15	12.00	2
LDHA	9.55	27.11	7
XRCC6	46.97	59.43	113
FASN	101.35	28.42	93
XRCC5	51.35	54.64	91
TOP2B	53.56	25.45	43
TUBA1A	19.25	47.88	37
TUBA1B	19.25	47.88	37
TUBB	17.3	40.6	35
TUBA4A	9.69	33.03	27
LMNB1	25.52	45.39	26
PRDX1	14.51	47.24	24
VCP	25.89	28.79	22
DDX3X	24.96	28.99	22
FLNA	38.19	14.43	21
SUPT16H	29.16	23.49	21
SSRP1	20.15	26.08	21
H2AFY	13.21	35.74	21
RBMX	12.75	32.73	21
TCP1	10.76	35.96	18
MDC1	25.5	12.02	16

Table S3. JTK-CYCLE analysis of quantitative protein expression.

	JTK_P	JTK_AMP	JTK_period
shSCR+Vector	0.087827048	0.326637491	24
shSCR+PRMT6	0.013512092	0.190028081	28
shPARP1+Vector	4.23E-09	0.592021553	20
shPARP1+PRMT6	0.007933384	0.308505038	24
shCUL4B+Vector	2.92E-06	1.099343063	24
shCUL4B+PRMT6	7.35E-05	0.654614384	28
shDDB1+Vector	7.97E-06	0.675017632	24
shDDB1+PRMT6	0.010390153	0.414673505	24
shSCR	0.010390153	0.306543341	24
shPRMT6	4.85009E-05	0.669102797	24
shPER3	1	0.073396339	24
shPRMT6+shPER3	0.00250066	0.32094188	28
Vector+DMSO	4.24415E-08	0.599567443	24
Vector+Olaparib	1.28163E-05	0.647867327	24
PRMT6+DMSO	1.71904E-06	0.333248593	28
PRMT6+Olaparib	0.013512092	0.506983322	28
shSCR+DMSO	1.71904E-06	0.668639062	24
shSCR+Olaparib	3.15869E-05	0.725306918	24
shPER3+DMSO	0.003377129	0.212793434	24
shPER3+Olaparib	0.001336632	0.686388454	28

Table S4. The information for the plasmid.

Plasmid	Expression	Tags	Promoter	Source
pCMV-tag2b-PRMT6	Mammalian	FLAG	CMV	This paper
pcDNA 3.0-3Flag-PARP1	Mammalian	FLAG	CMV	This paper
pGEX-4T-3-PRMT6	Bacterial	GST	Tac	This paper
pGEX-4T-3-PRMT6-N	Bacterial	GST	Tac	This paper
pGEX-4T-3-PRMT6-SAMD	Bacterial	GST	Tac	This paper
pGEX-4T-3-PRMT6-C	Bacterial	GST	Tac	This paper
pGEX-4T-3-PARP1	Bacterial	GST	Tac	This paper
pGEX-4T-3-PARP1-Z1	Bacterial	GST	Tac	This paper
pGEX-4T-3-PARP1-Z2	Bacterial	GST	Tac	This paper
pGEX-4T-3-PARP1-NLS	Bacterial	GST	Tac	This paper
pGEX-4T-3-PARP1-BRCT	Bacterial	GST	Tac	This paper
pGEX-4T-3-PARP1-CAT	Bacterial	GST	Tac	This paper
pGEX-4T-3-DDB1	Bacterial	GST	Tac	
pGEX-4T-3-DDB1-BPA	Bacterial	GST	Tac	
pGEX-4T-3-DDB1-BPB	Bacterial	GST	Tac	
pGEX-4T-3-DDB1-BPC	Bacterial	GST	Tac	
pGEX-4T-3-CUL4B	Bacterial	GST	Tac	
pGEX-4T-3-ROC1	Bacterial	GST	Tac	

Table S5. shRNA sequences.

shSCR	TTCTCCGAACGTGTCACGT
shPRMT6 #1	GGAGGGAACTGAAGAGGAAGA
shPRMT6 #2	GCCAGGTGAAGCAGCACTATG
shPRMT6 #3	GCAGCGCTTTGCTCAGCTAGA
shPER3 #1	GCTAAGGTGTATAATTGGATT
shPER3 #2	CGACAGCCTCTTCTGCGATAT
shPER3 #3	ATGACCATGAAGTTATCATTG
shPARP1	GGACCAAGTGTATGGTCAAGA
shCUL4B	GGATTCATTGGATAGCGTTCT
shDDB1	TCCACTAGATCGCGATAATAA

Table S6. The Primers used in quantitative real-time PCR (qPCR).

Gene	Strand	Sequence
PRMT6	F	TACCGCCTGGGTATCCTTCG
PRMT6	R	CCTGTTCCGGCAACTCTACA
PARP1	F	CGGAGTCTTCGGATAAGCTCT
PARP1	R	TTTCCATCAAACATGGGCGAC
CUL4B	F	GGCAACTGGAATAGAGGATG
CUL4B	R	TGTTCTTCAACCGTTTCTTTC
DDB1	F	ATCATCAAGCAAAGCACGATT
DDB1	R	ATCAAGGTATGTCAAGCACTC
BARD1	F	CTGCTCGCGTTGTACTAACAT
BARD1	R	TCCAATGCAGTCACTTACACAAT
ETAA1	F	ATGAGTCGGCGAAGGAAACAT
ETAA1	R	TCGGGGTTACTTTTACTGCAC
AXIN1	F	GACCTGGGGTATGAGCCTGA
AXIN1	R	GGCTTATCCCATCTTGGTCATC
ARID1B	F	GCAAGGTGTGAGTGGTTACTG
ARID1B	R	GGACTGGGACGGCAGATACT
EED	F	GTGACGAGAACAGCAATCCAG
EED	R	TATCAGGGCGTTCAGTGTTTG
TP53BP1	F	ATGGACCCTACTGGAAGTCAG
TP53BP1	R	TTTCTTTGTGCGTCTGGAGATT
CASP8	F	GTTGTGTGGGGTAATGACAATCT
CASP8	R	TCAAAGGTCGTGGTCAAAGCC
BCORL1	F	ATCTCTACAGCACCGCTCTAC
BCORL1	R	GTTGATGCCACACATGCGAAT
PRKCI	F	AGGTCCGGGTGAAAGCCTA
PRKCI	R	TGAAGAGCTGTTCGTTGTCAAA
PIK3CB	F	TATTTGGACTTTGCGACAAGACT
PIK3CB	R	TCGAACGTACTGGTCTGGATAG
AKT3	F	TGTGGATTTACCTTATCCCCTCA

AKT3	R	GTTTGGCTTTGGTCGTTCTGT
ETV1	F	CTGAACCCTGTAACTCCTTTCC
ETV1	R	AGACATCTGGCGTTGGTACATA
FYN	F	ATGGGCTGTGTGCAATGTAAG
FYN	R	GAAGCTGGGGTAGTGCTGAG
IDH1	F	TGTGGTAGAGATGCAAGGAGA
IDH1	R	TTGGTGACTTGGTCGTTGGTG
EGFR	F	AGGCACGAGTAACAAGCTCAC
EGFR	R	ATGAGGACATAACCAGCCACC
SETD1A	F	CAGTGGCGGAACTACAAGCTC
SETD1A	R	CATAGCGGTACACCTTCTGAGA
PER1	F	CCTACCCTCTCCCAGTGTTCTCTC
PER1	R	GGAACAGATAGTTAGGGAGCACCAAG
PER2	F	GTGAAGCAGGTGAAAGCCAATGAAG
PER2	R	CTCTCCATCTCCTCCACGGTGTAG
PER3	F	AGGTGAAGACAGAAAGCAAGAGAAGTG
PER3	R	GTGAGACAGCAAGGTTCCGATTCC
CSNK1A1	F	AGTGGCAGTGAAGCTAGAATCT
CSNK1A1	R	CGCCCAATACCCATTAGGAAGTT
BHLHE40	F	AGCAGTGGTTCTTGAACTTACC
BHLHE40	R	ACAAGCTGCGAAGACTTCAGG
CDC73	F	CTGGCCCAAGAATGTGAAGAC
CDC73	R	GCACGTCGGACATAAACAGGAT
ARID5A	F	CTGGCAAGCAGAACGGAATC
ARID5A	R	GCGTGTGTCGCTCCTTCAT
MEN1	F	GGGCTTCGTGGAGCATTTTCT
MEN1	R	GCGGCGATGATAGACAGGTC
EGR1	F	GGTCAGTGGCCTAGTGAGC
EGR1	R	GTGCCGCTGAGTAAATGGGA
FEXL3	F	AGTTTGCCGCAACTGGAAC
FEXL3	R	AGCTCTGGATGGGTAGCTTTC

MRE11	F	ATCGGCCTGTCCAGTTTGAAA
MRE11	R	TGCCATCTTGATAGTTCACCCAT
BAMI1	F	TGCCACCAATCCATACACAGAAGC
BAMI1	R	ATCTTCCCTCGGTCACATCCTACG
CLOCK	F	TCACATGCTGCGAGGAACAATA
CLOCK	R	ACCATTGTGCGCTGAAGAGGATAC
CRY1	F	TGGAAGTTGGATGTGGCTGTCTTG
CRY1	R	TCTCCATTGGGATCTGTTCTCCTACC
CRY2	F	TTCTACACGGCAGCTACCAACAAC
CRY2	R	GCATCAATCCAAGGGAAGCCTGTC
β-actin	F	AAGATCAAGATCATTGCTCCTC
β-actin	R	CATAGTCCGCCTAGAAGCA

Table S7. The Primers used in qChIP Assays.

Gene	Strand	Sequence
PER1	F	CTGGAGTTTGGCTTTCT
PER1	R	GAAGGATGCGGACAAT
PER3	F	AGCCGGGAGCCACCTA
PER3	R	GGAGGCTGGAAATGGG
CSNK1A1	F	GTCTCACCGCAACC
CSNK1A1	R	ACGCCTGTAATCCC
BHLHE40	F	GCGTTGTCCAACACGTG
BHLHE40	R	CGGTTAGGGCGGAGTG
CDC73	F	CAGCGTTTCAGCGAGTG
CDC73	R	AAGCACGTCCGCCATC
ARID5A	F	AGCCAATAGGAGTTCGT
ARID5A	R	TTGCCTAGCATCCCTC
MEN1	F	GCAAGATTCTGGCTCA
MEN1	R	GACTTCACGGGCTCAA
EGR1	F	AGGGTGCAGGATGGAGG
EGR1	R	CGGGGAACACTGAGAAGC
FEXL3	F	GTATTTAACTCCTCGGTTTC
FEXL3	R	GTCTCCTTGCTGTGGG
MRE11	F	CCTCGCCGCTACTTTC
MRE11	R	ACTGCTGGACTCTTCG
GAPDH	F	AGCCACATCGCTCAGACACC
GAPDH	R	CCCATACGACTGCAAAGACCC
